# Peptide Uptake Is Essential for *Borrelia burgdorferi* Viability and Involves Structural and Regulatory Complexity of its Oligopeptide Transporter

**DOI:** 10.1128/mBio.02047-17

**Published:** 2017-12-19

**Authors:** Ashley M. Groshong, Abhishek Dey, Irina Bezsonova, Melissa J. Caimano, Justin D. Radolf

**Affiliations:** a Department of Medicine, UConn Health, Farmington, Connecticut, USA; b Department of Pediatrics, UConn Health, Farmington, Connecticut, USA; c Department of Molecular Biology and Biophysics, UConn Health, Farmington, Connecticut, USA; d Department of Genetics and Genome Sciences, UConn Health, Farmington, Connecticut, USA; e Department of Immunology, UConn Health, Farmington, Connecticut, USA; MedImmune

**Keywords:** *Borrelia burgdorferi*, Lyme disease, nutrient limitation, oligopeptide, spirochete, transporter

## Abstract

*Borrelia burgdorferi* is an extreme amino acid (AA) auxotroph whose genome encodes few free AA transporters and an elaborate oligopeptide transport system (*B. burgdorferi* Opp [*Bb*Opp]). *Bb*Opp consists of five oligopeptide-binding proteins (OBPs), two heterodimeric permeases, and a heterodimeric nucleotide-binding domain (NBD). Homology modeling based on the crystal structure of liganded *Bb*OppA4 revealed that each OBP likely binds a distinct range of peptides. Transcriptional analyses demonstrated that the OBPs are differentially and independently regulated whereas the permeases and NBDs are constitutively expressed. A conditional NBD mutant failed to divide in the absence of inducer and replicated in an IPTG (isopropyl-β-d-thiogalactopyranoside) concentration-dependent manner. NBD mutants grown without IPTG exhibited an elongated morphotype lacking division septa, often with flattening at the cell center due to the absence of flagellar filaments. Following cultivation in dialysis membrane chambers, NBD mutants recovered from rats not receiving IPTG also displayed an elongated morphotype. The NBD mutant was avirulent by needle inoculation, but infectivity was partially restored by oral administration of IPTG to infected mice. We conclude that peptides are a major source of AAs for *B. burgdorferi* both *in vitro* and *in vivo* and that peptide uptake is essential for regulation of morphogenesis, cell division, and virulence.

## IMPORTANCE

*Borrelia burgdorferi*, the causative agent of Lyme disease, is an extreme amino acid (AA) auxotroph with a limited repertoire of annotated single-AA transporters. A major issue is how the spirochete meets its AA requirements as it transits between its arthropod vector and mammalian reservoir. While previous studies have confirmed that the *B. burgdorferi* oligopeptide transport (*opp*) system is capable of importing peptides, the importance of the system for viability and pathogenesis has not been established. Here, we evaluated the *opp* system structurally and transcriptionally to elucidate its ability to import a wide range of peptides during the spirochete’s enzootic cycle. Additionally, using a novel mutagenesis strategy to abrogate *opp* transporter function, we demonstrated that peptide uptake is essential for bacterial viability, morphogenesis, and infectivity. Our studies revealed a novel link between borrelial physiology and virulence and suggest that peptide uptake serves an intracellular signaling function regulating morphogenesis and division.

## INTRODUCTION

Amino acids (AAs) are essential for function and survival of the bacterial cell. In addition to providing the building blocks for protein synthesis, AAs are involved in peptidoglycan synthesis, nitrogen metabolism, energy generation, cell-cell communication, and environmental sensing (1, 2). Cellular AA homeostasis can be maintained by *de novo* biosynthesis, interconversion from other AAs or products of intermediary metabolism, and acquisition from the environment (3, 4). Free-living bacteria usually possess extensive biosynthetic pathways along with a large repertoire of free AA and/or di-, tri-, and oligopepeptide (Opp) transporters (5). Obligate pathogens, on the other hand, have varying capacities for *de novo* synthesis and interconversion, depending instead on repertoires of transporter evolutionarily tailored to exploit the range of nutrients available in the various niches that they inhabit (4, 6). Opp transporters are more energy efficient than single-AA transporters, enabling the import of multiple AAs per molecule of ATP (7).

The canonical Opp system consists of an oligopeptide-binding protein (OBP; OppA), a heterodimeric cytoplasmic membrane permease (OppBC), and a nucleotide-binding domain (NBD) heterodimer (OppDF) that drives transport by hydrolyzing ATP (7). In Gram-positive bacteria, OBPs are lipoproteins tethered to the external leaflet of the plasma membrane by N-terminal lipids and are capable of binding peptides as large as 35 AAs from the extracellular milieu, though only peptides of up to 18 AAs can be transported (7). In Gram-negatives, OBPs are periplasmic and nonlipidated and generally bind peptides of 2 to 5 AAs; the relatively small size of the peptide ligand is dictated by the dimensions of the porin channels through which they traverse the outer membrane permeability barrier (8). Although spirochetes have a double membrane ultrastructure resembling that of Gram-negatives, their OBPs are lipid modified as in Gram-positives, presumably to keep the binding protein in proximity to the permease to facilitate peptide import in these slowly growing organisms. According to structural data, OBPs contain an extended hinge region which facilitates binding of large substrates (7). The OBP binding cavity can accommodate a diverse range of peptides due to the presence of negatively charged residues lining their ligand binding pockets that interact with the peptide ligand backbone instead of specific AA side chains (9). However, some OBP homologues contain subtle structural modifications that allow binding of highly specific ligands (e.g., pheromones and muropeptides), which function as cues for the activation of signal transduction, cell competence, and gene regulation pathways (7). Conformational changes accompanying closure of the globular domains of the OBP around the ligand, a mechanism often described as a “Venus fly trap,” enable the OBP to dock to its cognate permease (7).

Bacterial Opp systems contain a great amount of variability with respect to gene arrangement and component multiplicity. *Escherichia coli* contains a canonical *opp* locus (*oppABCDF*) for support of nutrition along with *mppA* (murein peptide permease A), encoding an OppA-like OBP orphan that recycles muropeptides derived from the turnover of peptidoglycan via the Opp permease (see Fig. S1 in the supplemental material) (10). *Streptococcus pneumoniae* harbors a canonical *opp* locus (*amiA1BCDF*) as well as two separately located *oppA* genes (*aliA1* and *aliA2*) to import AAs for nutrition and the regulation of competence via the global regulator CodY (1, 11). *Lactococcus lactis* uses two separate ABC transporters, Opp and Opt, arranged in noncanonical gene order (*oppDFBCA* and *optSABCDF*), to support exponential growth from milk-derived peptides; OptS and OptA also transport milk-derived signaling peptides to regulate *opp* expression (12). *Staphylococcus aureus* contains four complete *opp* loci (*opp1ABCDF*, *opp2BCDF*, *opp3BCDFA*, and *opp4ADFBC*). Of these, only *opp3BCDFA* is required for uptake of peptides under AA-limiting conditions (6). The spirochete *Treponema denticola* has a complicated Opp system involving multiple OBPs, permeases, and NBD proteins arranged in noncanonical order (13). This oral commensal has limited AA biosynthetic capacity and instead uses an array of proteases to create a peptide-rich microenvironment in gingival tissues and energy generation via fermentation (14). In contrast, the syphilis spirochete, *T. pallidum*, an invasive, obligate human pathogen with almost no AA biosynthetic capability, contains an orphan OBP with no discernible cognate transporter, apparently relying on an assortment of AA ABC transporters and symporters to meet its AA requirements (15).

10.1128/mBio.02047-17.1 FIG S1  Genetic arrangements for bacterial Opp systems. OBPs, permeases, and NBD domains are designated in green, blue, and red, respectively. *B. recurrentis* contains a truncated oppA1* in which the codon for glutamine at position 44 has mutated to a stop codon. Download FIG S1, PDF file, 0.2 MB. Copyright © 2017 Groshong et al. 2017 Groshong et al. This content is distributed under the terms of the Creative Commons Attribution 4.0 International license.

**FIG 1:**
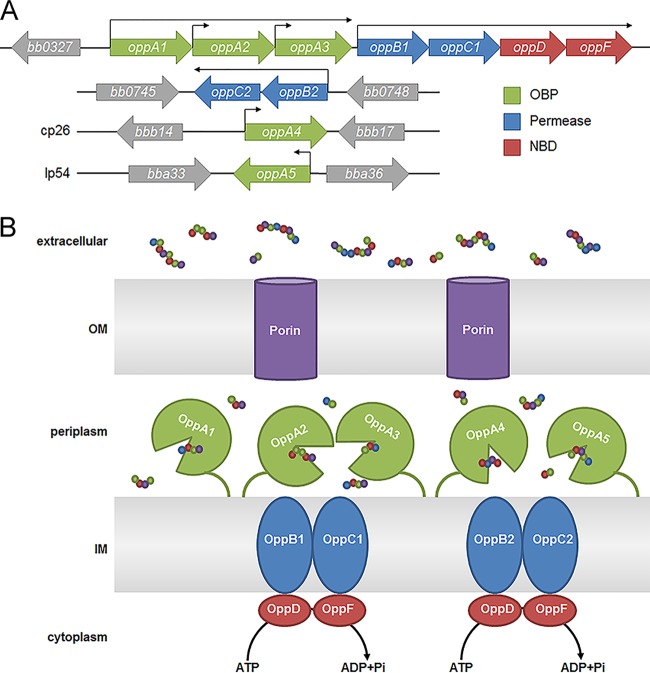
The *B. burgdorferi* oligopeptide transport system. (A) Genomic configuration with operonic and individual promoters designated by arrows (18, 30). (B) Working model. OM, outer membrane; IM, inner membrane.

*Borrelia burgdorferi*, the Lyme disease spirochete, must obtain essential nutrients from its arthropod vector and mammalian host (16, 17). Bioinformatics analyses reveal that *B. burgdorferi* contains only a few free AA transporters, no capacity for *de novo* AA synthesis, and can interconvert only serine and glycine (Table 1). Collectively, these data suggest that the spirochete’s elaborate Opp transport system (*B. burgdorferi* Opp [*Bb*Opp]) (Fig. 1) is a principal means by which the spirochete satisfies its AA requirements. *Bb*Opp consists of five OBPs (OppA1 to OppA5; three tandemly encoded in the chromosome and two encoded in separate plasmids), two heterodimer permeases (OppB1C1 and OppB2C2), and a heterodimer NBD domain (OppDF) (16, 18, 19). Since its first description by Bono et al. (18), studies have revealed that the *Bb*Opp system can import peptides when expressed in a heterologous host (i.e., *E. coli*), that the OBPs have overlapping specificities for small (3-to-7 AA) peptides (20, 21), and that differential expression of OBPs involves all of the spirochete’s known major gene regulatory pathways (Rrp2/RpoN/RpoS, Hk1/Rrp1, and Rel_Bb_) (18, 22–30). Infectivity studies performed with OBP transposon mutants have suggested that OppA2 (31) and OppA5 (32) are required for murine infection but that OppA1 is not (33). Nevertheless, despite this large body of work, we possess only a limited understanding of the contribution of this system to borrelial physiology. In this study, we utilized a combination of phylogenetics, structural biology, transcriptomics, and mutagenesis to develop a comprehensive understanding of how the individual Opp components function and of the overall importance of this system for spirochete survival *in vitro* and *in vivo*. Our approach has allowed us to identify unique as well as common features of the OBPs that help explain their collective ability to import a wide range of peptide substrates. By mutagenizing the ATP-hydrolyzing NBD domain to incapacitate the entire *Bb*Opp system, we demonstrated that peptides are essential for spirochete growth *in vitro*, as well as within the mammalian host, a milieu in which free AAs should be available (34). Most surprisingly, we found that spirochetes deprived of peptides via ablation of the Opp system not only failed to replicate but also displayed an elongated morphotype and dysregulated septation. Moreover, many elongated organisms lacked a planar waveform near mid-cell due to an absence of flagellar filaments, suggesting abnormalities in synthesis or insertion of new flagellar apparatuses near future division sites. We conclude that peptides are a major source of AAs for *B. burgdorferi* both *in vitro* and *in vivo* and that peptide uptake serves an intracellular signaling function regulating morphogenesis and cell division.

**TABLE 1:** *B. burgdorferi* amino acid transporters and enzymes

Gene	Designation	Predicted function^a^
Free amino acid transporters		
*bb0401*		Glutamate symporter
*bb0729*	*tcyP*	Cysteine transporter
*bb0637*	*nhaC* ortholog	Met, Tyr, or Lys transporter
*bb0638*	*nhaC* ortholog	Met, Tyr, or Lys transporter
*bb0843*	*arcD*	Arginine/ornithine symporter
		
Biosynthesis/interconversion enzymes		
*bb0841*	*arcA*	Arginine deiminase (Arg → citrulline)
*bb0842*	*arcB*	Ornithine carbamoyltransferase (citrulline → ornithine [PG])
*bb0160*	*alr*	Alanine racemase (L − Ala → d-Ala [PG])
*bb0601*	*glyA*	Serine hydroxyltransferase (serine ↔ glycine)

^a^ Function was predicted using CD-Search (99). PG, enzymes used for the generation of peptidoglycan.

## RESULTS

### The uniquely configured oligopeptide transport system of Lyme disease spirochetes.

As the starting point for our studies, we compared the configuration of borrelial Opp systems with those in phylogenetically diverse bacteria (see Fig. S1 in the supplemental material). The components of the Opp system of *B. burgdorferi* sensu lato are distributed among chromosome and plasmids (Fig. 1A and S1), mirroring the Lyme disease spirochete’s unusual, fragmented genome (35). Relapsing fever spirochetes transmitted by *Ornithodoros* soft ticks, and *B. miyamotoi*, a relapsing fever-like spirochete transmitted by the Lyme disease vector *Ixodes scapularis*, contain only *oppA1* and *oppA2* at the chromosomal *opp* locus harboring the other transporter components (Fig. S1) (36, 37). Remarkably, the louse-borne relapsing fever spirochete *B. recurrentis*, which is undergoing genome reduction from a *B. duttoni*-like precursor (38), has a mutation truncating OppA1 at AA 44 and thus employs only a single functional OBP (OppA2) throughout its life cycle (Fig. S1). All *Borrelia* spp. retain the secondary permease (OppB2C2; Fig. S1).

### Duplication and divergence of borrelial OppAs.

A dendrogram encompassing a wide range of OBPs shows that *Borrelia* OBPs form a distinct clade, with their closest relatives being OppAs of *T. denticola* (OppA5) and *T. pallidum* (Fig. S2A). In some bacterial species with multiple OBPs, such as *Borrelia* and *Brachyspira* species, the OBPs cluster on single nodes, suggesting that their respective genes arose by duplication. In contrast, the multiple OBPs in other species, such as *Bacillus anthracis*, *T. denticola*, and *Listeria monocytogenes*, are scattered around the tree, suggesting horizontal gene transfer. The *Borrelia* OBPs form two main branches (Fig. S2B), one containing OppA4 and OppA5, which form sister groups, and the second encompassing OppA1 to OppA3, with OppA2 occupying its own node. OppA1 and OppA2 are further segregated into Lyme disease and relapsing fever sister groups. From these relationships, we surmise that the Opp system of Lyme disease spirochetes and other borrelial species evolved from a series of gene duplications.

10.1128/mBio.02047-17.2 FIG S2  (A) Unrooted phylogenetic tree based on multiple-sequence alignments of OBPs from *B. burgdorferi* and other representative bacterial species. (B) A pruned tree showing only *Borrelia* species. Lyme disease spirochetes and relapsing fever spirochetes are shown in green and red, respectively, while other spirochetes are shown in blue. Download FIG S2, PDF file, 0.5 MB. Copyright © 2017 Groshong et al. 2017 Groshong et al. This content is distributed under the terms of the Creative Commons Attribution 4.0 International license.

### Structural features distinguish *Bb*OppA4 from Gram-negative and Gram-positive OBPs.

We took advantage of the crystal structure of *Bb*OppA4 (PDB ID: 4GL8) (39) to determine if *B. burgdorferi* OBPs possess structural elements compatible with promiscuous peptide binding, the functional hallmark of bacterial OBPs (40). The “closed” structure of *Bb*OppA4 (Fig. S3A), determined at a resolution of 2.2 Å, displays a well-defined, bilobed globular structure (domains II and III) with an extended hinge region (domain I) (7); the latter, a characteristic feature of “cluster C” substrate-binding proteins, provides extra space within the ligand binding cavity to accommodate peptides (5). Domain I of *Bb*OppA4 is formed from residues 30 to 62, 187 to 285, and 504 to 530 and contains seven centrally located β-sheets. Residues 63 to 186 form domain II, consisting of four stranded β-sheets and two α-helices. Residues 286 to 503 form domain III, which consists of four antiparallel β-strands sandwiched between three α-helices and two parallel β-strands with an α-helix. The final β-strand of domain III merges with the β-strand of domain I. In accord with the phylogenetic analysis, the lower Cα root mean square deviation (RMSD) values of *Bb*OppA4 with respect to *E. coli* OppA (*Ec*OppA) (1.27 Å) and *Salmonella enterica* OppA (*Se*OppA) (1.18 Å), compared to *L. lactis* OppA (*Ll*OppA) (6.57 Å), indicate that borrelial OBPs are structurally closer to their Gram-negative counterparts.

10.1128/mBio.02047-17.3 FIG S3  Comparison of *Bb*OppA4 structure to Gram-positive and Gram-negative OBPs. (A) Ribbon models of *Ec*OppA, *Se*OppA, *Ll*OppA, and *Bb*OppA4 showing domains I (purple), II (yellow), and III (green). (B) Ligand binding sites with interacting residues shown in stick representation, with dashed lines denoting hydrogen bonds. The conserved aspartic acid docks the N-terminal end of the ligand in *Ec*OppA (Asp445), *Se*OppA (Asp419), and *Bb*OppA4 (Asp431). (C) Hydrophobic docking site within the ligand binding cavity of *Ll*OppA. Residues lining the hydrophobic pocket are shown with a bold line. Hydrophobic residues are designated with asterisks (*); water molecules are blue, and the peptide ligand is red. Calculated volumes are shown below each binding cavity. (D) Configurations of binding cavities. Cavities are shown in red, with the X-Ala-Ala-Ala peptide shown in blue. (E) Electrostatic distribution of OBPs in the “open” conformation. “Open” *Bb*OppA4 was modeled against unliganded *Ec*OppA (PDB: 3TCH). Download FIG S3, PDF file, 0.4 MB. Copyright © 2017 Groshong et al. 2017 Groshong et al. This content is distributed under the terms of the Creative Commons Attribution 4.0 International license.

Fortuitously, the structure for *Bb*OppA4 was liganded to an endogenous X-Ala-Ala-Ala tetrapeptide (X denotes an N-terminal AA with unresolved electron density after the Cγ atom) in an extended conformation (39). *Bb*OppA4 contains a conserved Asp431 (Asp445 in *E. coli* and Asp419 in *S. enterica*) containing a side chain that hydrogen bonds to the amine group of the N-terminal residue of the peptide (Fig. S3B); this docking mechanism is characteristic of OBPs of Gram-negative bacteria (9). OppA from the Gram-positive *L. lactis*, in contrast, uses a large hydrophobic pocket in the ligand binding cavity to anchor a centrally located hydrophobic residue within the peptide (Fig. S3C) (40). *Bb*OppA4 contains a similar hydrophobic pocket which could accommodate a hydrophobic residue at position 2 in a bound peptide (Fig. S3C) and, therefore, could be a determinant of ligand specificity. In the crystal structure, *Bb*OppA4 engages its tetrapeptide via peptide backbone interactions (Fig. S3B). This finding indicates that *Bb*OppA4 is capable of sequence-independent binding and suggests that cavity size and geometry define the range of ligands. *Ec*OppA and *Se*OppA have the smallest binding cavities at 1,480 Å^3^ and 1,600 Å^3^, respectively, and are known to bind ligands of 2 to 5 AAs (Fig. S3C and D) (41). *Ll*OppA has the largest cavity at 4,785 Å^3^, consistent with its known capacity to bind and transport peptides as large as 18 AAs (7). The intermediate cavity size of *Bb*OppA4, 2,893 Å^3^ (Fig. S3C and D), suggests a capacity to bind peptides larger than tri- and tetrapeptides as shown elsewhere (21). Water molecules play a large role in determining OBP ligand size by filling empty space that can accommodate larger peptides and AA side chains. The presence of 26 water molecules within the cavity of *Bb*OppA4 (Fig. S3C) further suggests that larger peptides can be accommodated. Moreover, unlike *Ec*OppA and *Se*OppA, *Bb*OppA4 contains a water-filled extended region of the cavity near the conserved Asp with the potential to accommodate long-chain AAs or other bulky moieties at position 1 (Fig. S3C, right side of cavity).

The charge distribution within the ligand binding cavity can influence the peptide repertoire of a given OBP. To assess the electrostatics of the *Bb*OppA4 binding cavity, we modeled *Bb*OppA4 against the unliganded or “open” structure of *Ec*OppA (PDB ID: 3TCH). The *Ec*OppA cavity is primarily negatively charged and tends to prefer positively charged peptides (9, 42); *Bb*OppA4 is lined with positively charged residues, suggesting a preference for negatively charged peptides (Fig. S3E).

### Structural homology models explain diversity of peptide binding by *B. burgdorferi* OBPs.

**FIG 2:**
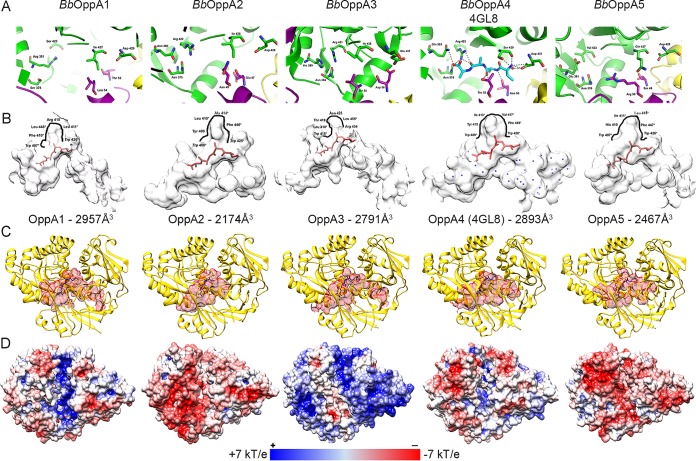
Homology models based on the *Bb*OppA4 structure explain diverse peptide binding by *B. burgdorferi* OBPs. Homology models for *Bb*OppA1, *Bb*OppA2, *Bb*OppA3, and *Bb*OppA5 were generated using the *Bb*OppA4 template (39) and SWISS-MODEL (Cα RMSD values, 0.05 to 0.07 Å). (A) Ligand binding sites. The *Bb*OppA4 structure includes the bound endogenous peptide (X-Ala-Ala-Ala), with dashed lines denoting hydrogen bonds; a conserved aspartic acid (Asp431) docks the N-terminal end of the peptide. For homology models, ligand binding sites are shown without ligands and interacting residues are shown as stick representations. (B) Ligand binding cavities containing the X-Ala-Ala-Ala peptide (in red) were defined using the CASTp server and a 1.4-Å probe. Water molecules in the binding cavity of *Bb*OppA4 are shown in blue. Residues lining the pockets in proximity to residue 2 of the X-Ala-Ala-Ala peptide are indicated with bold lines; hydrophobic residues lining the pocket are noted with an asterisk (*). Calculated volumes are shown below each binding cavity. (C) Configurations of binding cavities. Cavities are shown in red, with the X-Ala-Ala-Ala peptide shown in blue. (D) Electrostatic distribution of *B. burgdorferi* OBPs in the “open” conformation. OBPs were modeled against unliganded *E. coli* OppA (PBD: 3TCH); electrostatics were calculated using the APBS server.

Using the *Bb*OppA4 structure as a template, we created *in silico* structural homology models of the other *B. burgdorferi* OBPs to understand how variation in the OBP sequences could broaden the peptide ligand repertoire of the spirochete’s Opp system. RMSD values based on Cα deviations (0.05 to 0.07 Å) for the homology models demonstrate strong structural similarity to *Bb*OppA4 (Fig. S4A). Four *B. burgdorferi* OBPs contain the conserved N-terminal docking aspartate (*Bb*OppA1, Asp429; *Bb*OppA2, Asp428; *Bb*OppA4, Asp431; *Bb*OppA5, Asp429), while *Bb*OppA3 contains a glutamic acid at position 437, which would function analogously (Fig. 2A and S4B). The multiple-sequence alignment of *B. burgdorferi* OBPs (Fig. S4C) demonstrates that the putative ligand binding residues of the modeled OBPs align with the binding residues identified in the *Bb*OppA4 structure. The projection of the *Bb*OppA4 ligand into the *Bb*OppA2 and *Bb*OppA3 binding sites shows the greatest conservation of predicted ligand-interacting residues across the five OBPs, while *Bb*OppA4, *Bb*OppA1, and *Bb*OppA5 contain one, two, and three unique substitutions, respectively, within their known or putative ligand-binding cavities. The variable residues are situated at different contact points along the peptide; *Bb*OppA1 contains substitutions along the linear edge of the peptide, while *Bb*OppA4 has a substitution at the C-terminal end of the peptide. There is variability in the distribution of hydrophobic residues along the pockets near residue 2 of the *B. burgdorferi* OBPs, suggesting a dichotomy with respect to the preferred residue(s) at position 2 of the liganded peptide (Fig. 2B). *Bb*OppA2, *Bb*OppA4, and *Bb*OppA5 are predicted to accommodate bulky hydrophobic residues at this position, while *Bb*OppA1 and *Bb*OppA3 may accept both large hydrophobic and polar side chain residues. The volumes of the *B. burgdorferi* OBP binding cavities range from 2,174 Å^3^ to 2,957 Å^3^ (Fig. 2C and D). The smallest of these, *Bb*OppA2, has been shown to bind heptapeptides (21), suggesting that all *B. burgdorferi* OBPs can minimally bind peptides of seven AAs. The extended region near the conserved aspartate of *Bb*OppA4 is present in *Bb*OppA1, *Bb*OppA3, and *Bb*OppA5, but not *Bb*OppA2 (Fig. 2B, right side of cavity). Modeling of the *B. burgdorferi* OBPs using the open conformation of *Ec*OppA reveals variability in electrostatic distributions within the ligand binding cavity (Fig. 2D). The binding cavity of *Bb*OppA1, like that of *Bb*OppA4, is positively charged, while those of *Bb*OppA2 and *Bb*OppA5 are negatively charged. *Bb*OppA3 is unique, presenting a neutral binding cavity. Projecting the AA variability of *Bb*OppA1 to *Bb*OppA5 onto the *Bb*OppA4 structure, we find that the highest variability is within the ligand binding region, likely reflected by the variation in electrostatics, while the outer surface regions are more highly conserved (Fig. S5). Taken together, these comparisons strongly argue that the *B. burgdorferi* OBPs are not functionally redundant.

10.1128/mBio.02047-17.4 FIG S4  Validation of homology models of *B. burgdorferi* OBPs against *Bb*OppA4 structure. (A) Superimposed images of each *B. burgdorferi* OppA homology model against the *Bb*OppA4 crystal structure, with RMSD values based on Cα deviations. (B) Ligand binding pockets with interacting residues shown in stick representation, with dashed lines denoting potential hydrogen bonds with X-Ala-Ala-Ala. (C) Multiple-sequence alignment of *B. burgdorferi* OBPs showing THE *Bb*OppA4 secondary structure above sequences; ligand binding residues are denoted by asterisks (*) below sequences. Amino acid identity is denoted by red boxes, and similarity in a group is denoted by red characters; similarity across groups is denoted by blue frames, and gaps are denoted by a period. Download FIG S4, PDF file, 0.6 MB. Copyright © 2017 Groshong et al. 2017 Groshong et al. This content is distributed under the terms of the Creative Commons Attribution 4.0 International license.

10.1128/mBio.02047-17.5 FIG S5  Evolutionary conservation of *B. burgdorferi* OBPs by ConSurf analysis. A ribbon model of *Bb*OppA4 colored by conservation of AAs among *Bb*OppA1-5 using variability color coding, with ligand shown in black and conserved Asp in stick representation, is presented. Download FIG S5, PDF file, 0.1 MB. Copyright © 2017 Groshong et al. 2017 Groshong et al. This content is distributed under the terms of the Creative Commons Attribution 4.0 International license.

Closure of an OBP around its ligand creates a docking site for the cognate permease. The *Bb*OppA2, *Bb*OppA4, and *Bb*OppA5 surface domains present predominantly negative surface charges, while the surfaces of *Bb*OppA1 and *Bb*OppA3 are more neutral and positively charged, respectively (Fig. 2D). These results suggest that the *B. burgdorferi* OBPs have differing affinities for the two permeases.

### Differential expression of *opp* genes throughout the enzootic cycle.

**FIG 3:**
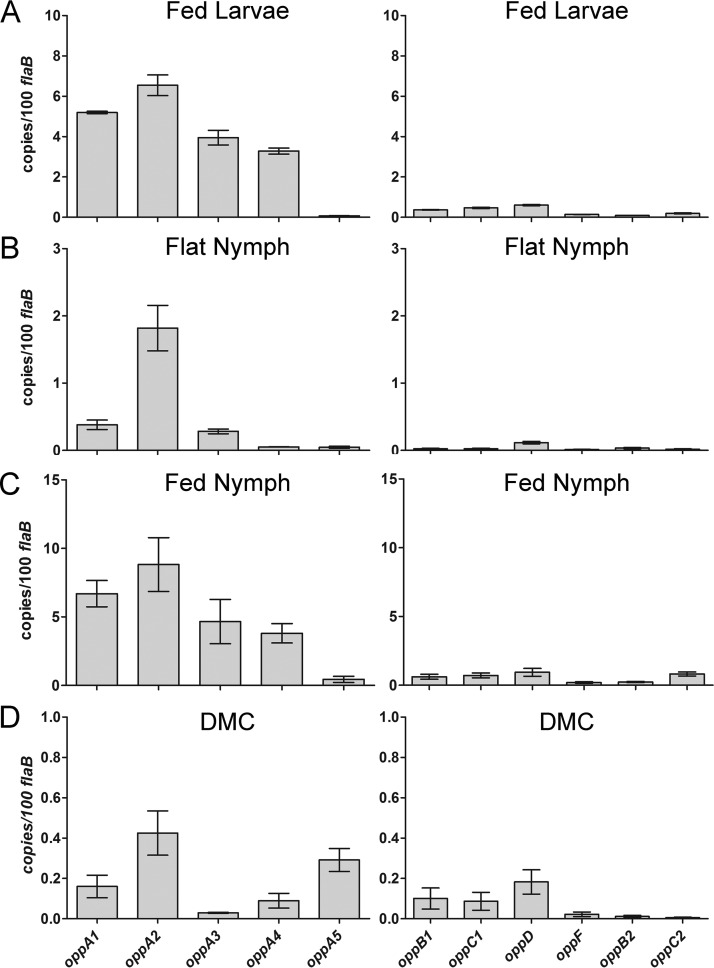
Differential expression of *opp* genes throughout the enzootic cycle. Data represent results of qRT-PCR analysis of transcripts from (A) fed larvae, (B) flat nymphs, (C) fed nymphs, and mammalian-host-adapted spirochetes cultivated in dialysis membrane chambers (DMCs) (D). Note that for spirochetes from unfed ticks and DMCs, the *y* axis is expanded because of the lower transcript copy numbers. Values represent averages of results from experiments performed with three biological samples, each done in quadruplicate. Error bars indicate standard errors of the means. *P* values for pairwise comparisons, determined using a two-tailed *t* test, are provided in Table S1.

While the expression profiles of individual *opp* genes have been examined previously (23–28, 30), regulation of the system throughout the enzootic cycle remains poorly understood. To investigate this, we used quantitative reverse transcriptase PCR (qRT-PCR) to compare the transcript levels for all 11 *opp* genes in flat and feeding ticks and within the mammal using our dialysis membrane chamber (DMC) model (Fig. 3). Not surprisingly, spirochetes displayed a unique *opp* profile under each condition. As in other bacterial species (7), *B. burgdorferi* oppAs are transcribed at relatively high levels compared to other transporter components, presumably to provide the permeases with a steady stream of cargo. Previously, Bono et al. (18) and Wang et al. (30) reported that *oppA1* to *oppA3* can be transcribed as an operon or individually during *in vitro* cultivation. We detected relatively similar transcript levels for all three genes in fed larvae and nymphs, consistent with operonic transcription (Fig. 3A and B), but substantially higher levels of *oppA2* in flat nymphs and DMCs, suggesting individual regulation (Fig. 3B and D). Expression of the plasmid-borne OBPs was host specific, with *oppA4* expressed only in feeding ticks (Fig. 3A and C) and *oppA5* expressed only in mammals (Fig. 3D).

10.1128/mBio.02047-17.6 TABLE S1  *P* value matrix for expression analysis using a two-tailed *t* test. Download TABLE S1, DOCX file, 0.03 MB. Copyright © 2017 Groshong et al. 2017 Groshong et al. This content is distributed under the terms of the Creative Commons Attribution 4.0 International license.

### Loss of Opp transporter function abrogates spirochete growth *in vitro*.

**FIG 4:**
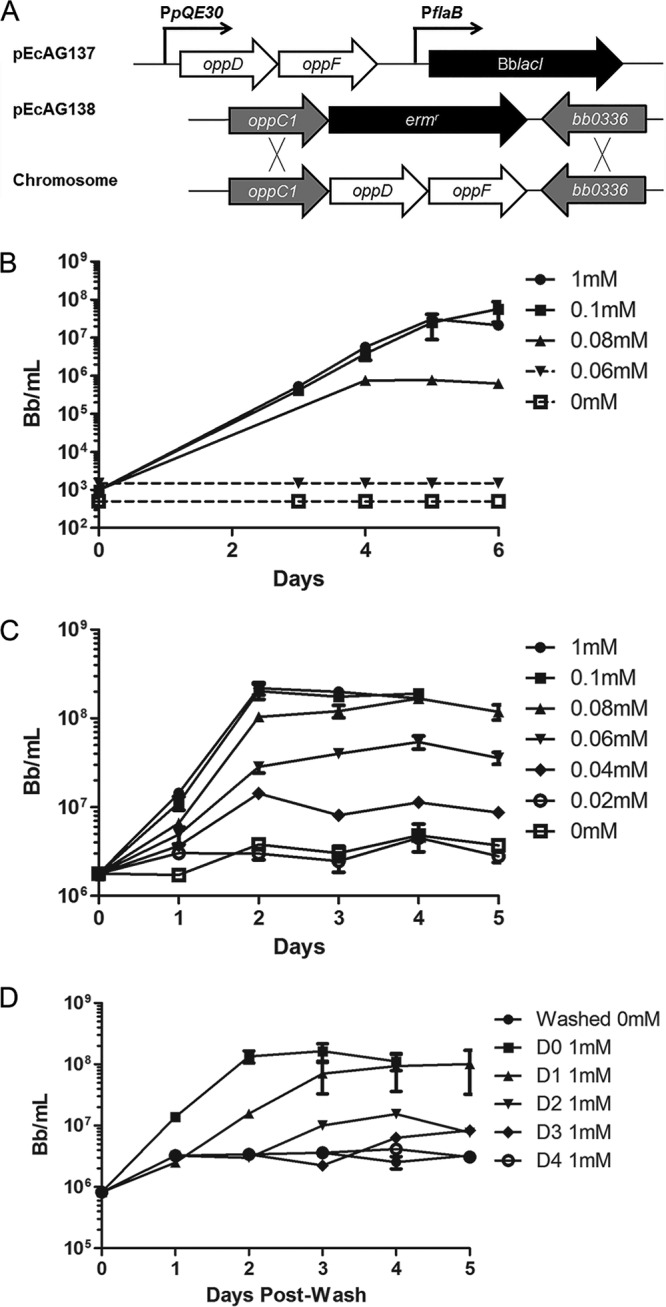
Loss of Opp transporter function abrogates spirochete growth *in vitro*. (A) Schematic showing construction of the conditional NBD mutant (BbAG132). (B) Growth curves showing the effects of IPTG titration on a low starting inoculum (1 × 10^3^ spirochetes/ml) of the NBD mutant followed for 6 days in BSK-II medium. (C) Growth curves showing the effects of IPTG titration at a high starting inoculum (1 × 10^6^ spirochetes/ml) of the NBD mutant followed for 5 days. (D) Growth curves showing the ability of IPTG-starved cultures to recover following the addition of IPTG. The mutant, grown to mid-logarithmic phase with 1 mM IPTG, was washed and inoculated into 15 ml of BSK-II (~1 × 10^6^ spirochetes/ml) without IPTG; 1 mM IPTG was added to starved cultures at daily intervals, following which growth was monitored by dark-field microscopy for 5 days. In all experiments, growth curve analyses were performed in triplicate. Dotted lines denote cultures with densities below the limits of detection by dark-field microscopy.

While previous studies have confirmed that the *B. burgdorferi* oligopeptide transport (*opp*) system is capable of importing peptides (20, 21), the importance of the system for spirochete viability and pathogenesis has not been established. We devised a genetic strategy to address this issue—deletion of the heterodimeric NBD domain (*oppDF*) to deprive the Opp system of its energy source. Repeated attempts to create *oppDF* deletion mutants were unsuccessful, suggesting that the *Bb*Opp system is essential. As an alternative approach, we generated a conditional NBD mutant (BbAG132) in which expression of *oppDF* can be induced with IPTG (isopropyl-β-d-thiogalactopyranoside) (Fig. 4A). To ensure tight repression, we utilized the IPTG-inducible construct developed by Blevins et al. (43), which contains two *lac* operator sequences. The mutant was grown in Barbour-Stoenner-Kelly II medium (BSK-II) supplemented with IPTG (0 to 1 mM) using starting inocula of 1 × 10^3^ spirochetes/ml, and triplicate cultures were enumerated for 6 days by dark-field microscopy (Fig. 4B). No growth was observed at IPTG concentrations below 0.08 mM. At 0.08 mM IPTG, there was a noticeable lag in growth, and spirochetes failed to reach peak density. At higher IPTG concentrations, organisms grew at wild-type levels. We next repeated the growth curves with higher inocula (~1 × 10^6^ spirochetes/ml) to allow enumeration of spirochetes throughout the period of incubation. We saw no replication at concentrations below 0.04 mM IPTG and a noticeable lag in growth with substantially lower peak densities at 0.04 and 0.06 mM IPTG (Fig. 4C). Last, we tested whether the addition of IPTG to uninduced mutant cultures would restore growth (Fig. 4D). Spirochetes were initially cultured to mid-logarithmic phase with 1 mM IPTG, washed, and resuspended in BSK-II lacking IPTG. Addition of 1 mM IPTG on day 0 and day 1 after resuspension restored normal growth. Growth following addition of IPTG on day 2 was suboptimal, while addition of IPTG at later time points did not restore replication.

### Peptide starvation induces dramatic morphological changes.

**FIG 5:**
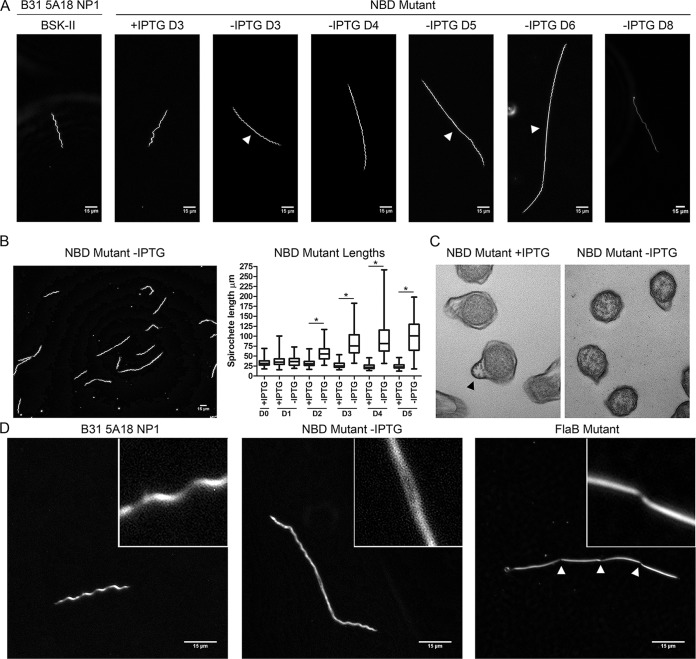
Peptide starvation induces dramatic morphological changes during *in vitro* cultivation. (A) Results of dark-field microscopy (magnification, ×400) showing the wild-type strain (B31 5A18 NP1), the NBD mutant with (+) IPTG and without (-) IPTG. D3 of the wild-type strain and NBD mutant with IPTG is representative of all time points. Arrowheads designate central flattening in mid-cell regions of the NBD mutant without IPTG. (B) Representative image (magnification, ×400) of the conditional NBD mutant without IPTG on D4 post-depletion along with whisker plots of spirochete lengths measured using the Metamorph integrated morphometry analysis tool. Asterisks (*) denote *P* values of <0.0001 for pairwise comparisons at each time point determined using a two-tailed *t* test. (C) Transmission electron microscopy cross sections demonstrating diminished flagellation in organisms incubated without IPTG. Flagellar bundles are denoted with an arrowhead. (D) Dark-field microscopy (magnification, ×1,000) of the wild-type strain, a conditional NBD mutant without IPTG (D5), and a Δ*flaB* mutant. Septal invaginations in the Δ*flaB* mutant are denoted with arrowheads.

Notably, NBD mutants grown without IPTG progressively elongated (Fig. 5A), with considerable heterogeneity in the degree of elongation at each day of observation (Fig. 5B). From day 2 onward, the differences in lengths between induced and uninduced mutants, measured using the Metamorph integrated morphometry analysis tool, were highly significant (*P* ≤ 0.0001, Fig. 5B). By day 8, mutants grown without IPTG were nonmotile, and some showed a semitranslucent “ghostlike” appearance, indicative of nonviability (Fig. 5A, -IPTG D8). While some elongated spirochetes were helical along their entire lengths, others demonstrated loss of helicity near mid-cell (Fig. 5A). Spirochetes in the latter group displayed motility at the cell poles but propagated waveforms weakly or not at all toward the cell centers (see Movies S1 and S2 in the supplemental material). The planar wave morphology of *B. burgdorferi* arises from the force exerted by rigid periplasmic flagellar filaments against the elastic peptidoglycan sacculus (44). We used transmission electron microscopy to determine whether the central flattening was due to the absence of flagella. Similar to Δ*flaB* spirochetes (45), cultures of the NBD mutant without IPTG contained numerous cross sections without outer membrane protrusions containing flagellar bundles (Fig. 5C). Regardless of whether a waveform was present, elongated mutants lacked discernible septal invaginations. We confirmed this by comparison with Δ*flaB* spirochetes, which septate but do not separate (45) (Fig. 5D). Elongated NBD mutant spirochetes that retained a planar waveform oscillated in segments of approximate wild-type length (see Movie S3). Together, these data confirm that peptide starvation results in gross morphological anomalies and perturbation of normal cell division events.

10.1128/mBio.02047-17.8 MOVIE S1  Motility of wild-type spirochete. All movies were acquired at a magnification of ×400 for 20 frames for a total duration of 2.755 s (0.13775 s per frame). Video frame rates were slowed to 10 frames/s using AVI FR Changer v1.10 (Inmatrix; http://inmatrix.com/files/avifrate_download.shtml). The videos can be viewed using Windows Media Player or VLC media player for Mac OS X (available at http://www.videolan.org/vlc/download-macosx.html). Download MOVIE S1, AVI file, 2.4 MB. Copyright © 2017 Groshong et al. 2017 Groshong et al. This content is distributed under the terms of the Creative Commons Attribution 4.0 International license.

10.1128/mBio.02047-17.9 MOVIE S2  Motility of starved NBD mutant with central flattening. Download MOVIE S2, AVI file, 4.4 MB. Copyright © 2017 Groshong et al. 2017 Groshong et al. This content is distributed under the terms of the Creative Commons Attribution 4.0 International license.

10.1128/mBio.02047-17.10 MOVIE S3  Motility of starved NBD mutant with full waveform. Download MOVIE S3, AVI file, 3.8 MB. Copyright © 2017 Groshong et al. 2017 Groshong et al. This content is distributed under the terms of the Creative Commons Attribution 4.0 International license.

### Peptide uptake is essential for viability, morphogenesis, and virulence within the mammalian host.

**FIG 6:**
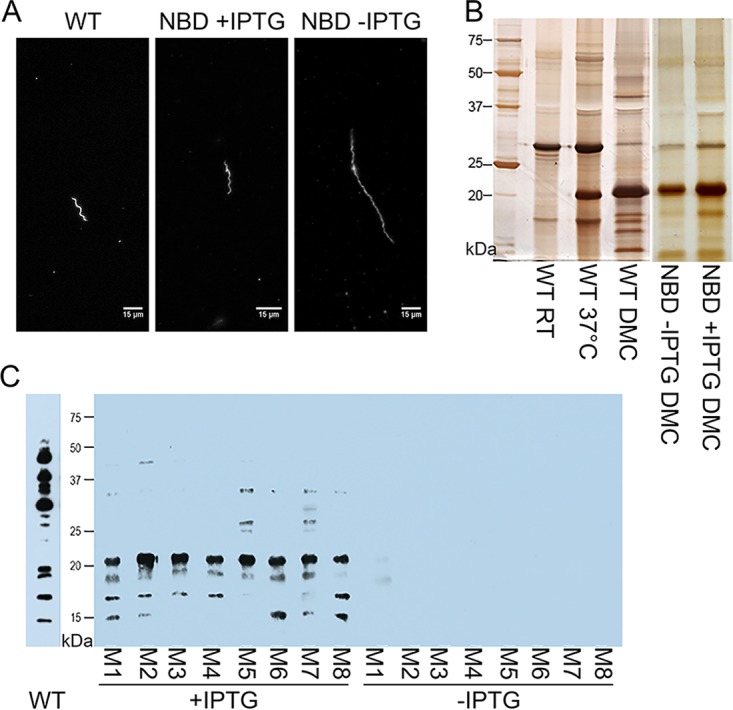
Peptide uptake is essential for viability, morphogenesis, and virulence within the mammalian host. (A) Dark-field microscopy (magnification, ×400) demonstrates cell morphology of the conditional NBD mutant compared with wild-type spirochetes in DMC chambers. (B) Silver stain of SDS-PAGE gel for the wild-type strain and the conditional NBD mutant (with and without IPTG) from DMC explants. (C) Immunoblotting of *B. burgdorferi* whole lysate using sera from mice with and without IPTG supplementation at 2 weeks after needle inoculation with the NBD mutant. An immunoblot of serum from a mouse two weeks after inoculation with wild-type spirochetes is shown alongside. Blots represent two independent experiments, each performed with two cohorts of 4 mice each (Exp 1, M1 to M4; Exp 2, M5 to M8). Molecular markers are shown for the gel and Western blot in kilodaltons.

We used the DMC system to assess the importance of the Opp transporter for viability within the mammal. Chambers inoculated with 1 × 10^4^ wild-type or NBD mutant spirochetes/ml (gently washed to remove residual IPTG) were implanted for 2 weeks. Wild-type organisms grew normally and were host adapted (Fig. 6A and B), while no NBD mutants were visualized by dark-field microscopy. We next determined whether peptide-starved mutants cultivated in DMCs develop morphological abnormalities comparable to those observed *in vitro*. To ensure a sufficient density of organisms for evaluation by microscopy, we modified our standard protocol by (i) using a 10-fold-greater inoculum (1 × 10^5^ *B. burgdorferi* spirochetes/ml); (ii) inoculating DMCs with NBD mutant organisms cultured *in vitro* to mid-logarithmic phase with 1 mM IPTG; and (iii) explanting DMCs at an early time point (7 days) when viable organisms are more likely to be present. Spirochetes explanted from rats given IPTG-supplemented water appeared morphologically similar to wild-type spirochetes, while organisms recovered from animals given untreated water were markedly elongated, although with normal waveforms (Fig. 6A). SDS-PAGE revealed that the DMC-cultivated spirochetes host adapted with or without IPTG supplementation (Fig. 6B).

Last, we assessed whether the Opp system is required for infectivity. Three days prior to syringe inoculation, we began administering IPTG-supplemented water to C3H/HeJ mice (*n* = 8). On day 0, IPTG-treated and untreated mice were subjected to needle inoculation with NBD mutants (1 × 10^4^ spirochetes). For both groups of mice, cultures of the inoculation site, ear, tibiotarsal joint, and heart collected 2 weeks postinoculation in BSK-II containing IPTG were negative. However, whereas sera from NBD-infected mice given plain water displayed no immunoreactivity, all eight mice given IPTG prior to and during infection seroconverted (Fig. 6C).

## DISCUSSION

In most bacteria, Opp systems are ancillary and used to supplement pathways for biosynthesis of AAs and mechanisms for procuring them from the environment. The Lyme disease spirochete is an extreme AA auxotroph whose limited repertoire of AA transporters is unable to provide a full complement of these essential nutrients. Previous investigators surmised that the *Bb*Opp system could play a central role in maintaining borrelial homeostasis (18, 20, 21, 30). While facets of the *Bb*Opp system have been explored (18, 20, 21, 27, 31–33), its essentiality has never been demonstrated, nor have strategies been developed to clarify how its various components functionally interrelate. In this study, we confirmed that, even with the capacity to transport some free AAs, *B. burgdorferi* requires peptides for growth *in vitro*, within the mammal and, presumably, within the tick. Moreover, we show that the *B. burgdorferi* Opp system possesses a modular framework that enables adaptation to changing microenvironments via a combination of differential *oppA* expression and variations on the structural theme of sequence-independent peptide binding, the functional hallmark of prototypical OBPs (40).

Bacteria often encode multiple OBPs as a means of exploiting the full range of peptides available in a given niche (7); OBP diversity can be achieved by lateral gene transfer and/or gene duplication with subsequent sequence variation. Our phylogenetic and structural analyses, taken together, suggest that OBP diversity in *Borrelia* spp. arose by a combination of gene duplication events and mutations within domains II and III that modified the geometry and electrostatics of the ligand binding cavities as well as the surface residues that form the permease docking sites. Hu and colleagues (20, 21) established an approximate size range (3 to 7 AAs) of the peptides bound by *B. burgdorferi* OBPs and demonstrated that individual *B. burgdorferi* OBPs have both overlapping and distinct specificities for peptide substrates. Given the combinatorial complexity of peptide-binding studies involving all five *B. burgdorferi* OBPs, we reasoned that a structural approach would inform our understanding of the binding preferences for individual *B. burgdorferi* OBPs as well as of their collective ability to meet the spirochete’s AA requirements. The structure of *Bb*OppA4 confirmed that, as with other bacterial OBPs (9), *B. burgdorferi* OBPs achieve ligand promiscuity by binding to peptide backbones. Then, using the *Bb*OppA4 structure as a template, we demonstrated that modifications in cavity binding parameters, such as volume, configuration, and electrostatics, likely determine the ligand preferences of each OBP. Particularly striking is the variation in electrostatics of the binding cavities, implying that peptides are bound or excluded on the basis of their overall charge. We surmise, therefore, that each OBP accommodates a unique array of peptides that collectively enable the spirochete to meet its AA needs throughout the enzootic cycle, including the microenvironments that it occupies to persist within the mammal. We also noted differences in the surface electrostatics of the permease docking sites, most notably for *Bb*OppA3, that likely alter the ability of a given OBP to compete for access to either (or both) permease(s).

As seen in previous studies (22–28), differential regulation of the *Bb*Opp system primarily involves modulation of *oppA* gene expression. This transcriptional versatility derives, in large part, from the modular organization of the transporter genes within the *B. burgdorferi* genome. The expression profiles of *oppA4* and *oppA5*, orphan OBPs encoded on separate genetic elements, represent excellent cases in point. *oppA4* is expressed during the larval and nymphal blood meals and has been shown to be positively regulated by c-di-GMP, a signaling molecule generated by the Hk1/Rrp1 two-component system active in the feeding tick (23). *oppA5*, on the other hand, is absolutely RpoS dependent but is also repressed by c-di-GMP; these results, taken together, may explain why this gene is expressed exclusively in mammals (23, 24, 27, 28). Among the five *B. burgdorferi* OBPs, OppA5 has the most divergent ligand binding cavity with respect to ligand binding residues, suggesting a distinct functionality during mammalian infection. The three chromosomally encoded OBPs are organized as an operon but exhibit expression profiles that clearly are not simply polycistronic. Bono et al. (18) identified mono-, bi-, and tricistronic transcripts by Northern blot analysis, while Wang et al. (30) confirmed that each *Bb*OppA gene has its own promoter. *oppA1* to *oppA3* are expressed in the feeding tick at comparable levels, whereas only *oppA2* is highly expressed in flat ticks and DMCs. A potential regulator of this locus is the alarmone (p)ppGpp, a product of Rel_Bb_ which has been shown to modulate expression of *oppA1*, *oppA2*, and *oppA3* and to be required for survival of spirochetes in ticks (22, 25). Last, our finding that OppA2 is expressed at relatively high levels throughout the enzootic cycle strongly implies that this OBP is a functional centerpiece of *Bb*Opp. This inference is buttressed by the fact that OppA2, whose ligand binding cavity features are highly conserved among *Borrelia* species, is the only full-length OBP in the louse-borne relapsing fever spirochete *B. recurrentis*. Furthermore, a transposon mutant for *oppA2*, but not *oppA1*, is attenuated for murine infection (31, 33).

In recent years, investigators have made considerable inroads elucidating *B. burgdorferi*'s mechanisms for carbon uptake and their regulation as carbon sources change during the enzootic cycle (31, 46–54). On the other hand, AA uptake by the spirochete has received much less attention and, not surprisingly, is much more poorly understood. Using a state-of-the-art mutagenesis technique (43, 55, 56) to target OppDF, the energy lynchpin of the Opp permease, we definitively demonstrated that peptides are essential for replication of *B. burgdorferi in vitro* and *in vivo* and that the Opp system is the principal if not the only means by which the bacterium can acquire them. Indeed, we saw a direct correlation between the concentration of IPTG used for induction of the NBD domain and spirochete replication *in vitro*. The relatively low expression levels of the NBD domains, determined by qRT-PCR analysis, likely explain why the growth rate of the constitutive mutant was exquisitely sensitive to small changes in the IPTG concentration. That limited replication occurred at a minimal concentration of IPTG (i.e., 0.04 mM) suggests a low threshold for stimulation of cell division by cytosolic peptides and/or AA pools.

Unexpectedly, in the absence of IPTG, cells not only ceased to replicate but progressively elongated, and wild-type growth patterns could not be restored following a relatively short period of cultivation without inducer. Thus, in contrast to the adaptive slowing of replication during peptide limitation, peptide starvation causes irreversible dysregulation of morphogenesis and cell division, a complex set of responses that are obviously maladaptive and not easily explained given our current knowledge of *B. burgdorferi* cell biology, physiology, and gene regulation. Residual NBD may enable some peptide transport during the initial period of cultivation without IPTG. A recent study using an inducible FtsH, an ATP-dependent cytoplasmic membrane-bound protease, demonstrated that spirochetes had to be cultured for 48 h without inducer for full degradation of preexisting protein (55). Even with complete cessation of peptide uptake, limited protein synthesis could be supported by uptake of some free AAs by dedicated transporters, “cannibalization” of preexisting proteins, and redirection of protein synthesis away from nonessential polypeptides. A striking feature of the peptide starvation phenotype is the inhibition of septum formation. Septum formation has long been known to be triggered by signals involving protein synthesis (57). More recently, direct links have been established between carbon and nitrogen metabolism and Z-ring formation (58, 59). Thus, it is conceivable that depletion of intracellular peptide and/or AA pools results in failure to provide one or more of the metabolic signals essential for divisome assembly. Another fascinating aspect of the morphotype is the heterogeneity in helicity of the elongated organisms, which could be attributed to the presence or absence of flagellar filaments near mid-cell. Separation of dividing spirochetes requires motive force provided by newly assembled flagellar apparatuses flanking the division site (45). Perhaps some elongated spirochetes become “arrested” in a preseptation stage of the cell cycle such that new flagellar motors are not inserted (60). A diminished capacity to synthesize flagellar motor and filament proteins is likely also contributory. Experiments are under way to define the dysregulated steps in divisome and flagellar assembly and how they relate to the metabolic perturbations caused by peptide depletion. Given the poor growth and elongation of mutants cultivated in DMCs implanted into rats not given IPTG, we surmise that the same phenotype explains the avirulence of the NBD mutant and that incomplete complementation reflects the difficulty in maintaining adequate levels of IPTG by *ad libitum* oral administration, limited penetration of IPTG into tissues, and/or or suboptimal dosing.

The major implication of our study is that peptides must be available to support spirochete metabolism throughout the enzootic cycle. During both the larval and nymphal blood meals, when spirochetes undergo rapid expansion (61), salivary proteases produce a glut of peptides from host proteins, such as serum albumin and hemoglobin (62, 63). As the molt progresses and spirochetes become quiescent, protein homeostasis is likely maintained by residual blood meal components (64). Our demonstration that peptides are an absolute requirement for *B. burgdorferi* in the mammal presents a conundrum regarding the source of the peptides, particularly within the noninflammatory milieu associated with persistence in the reservoir host, *Peromyscus leucopus* (65). The literature provides little to no evidence for the presence of free peptides in blood and interstitial fluid at concentrations presumably needed for bacterial replication. One can envision two complementary mechanisms by which *B. burgdorferi* could generate the requisite peptides. One is by secreting an extracellular protease, such as HtrA (66, 67). The other is by decorating its surface with host-derived proteolytic enzymes, such as plasminogen and urokinase-type plasminogen activator (68, 69). The paucibacillary nature of Lyme disease is well recognized and is the root cause for many of the diagnostic dilemmas associated with *B. burgdorferi* infection (70). It is tempting to speculate that the ability of *B. burgdorferi* to scavenge peptides, a physiological cornerstone of its persistence strategy, also serves as a novel form of *in vivo* growth regulation that facilitates its ability to “fly below the radar” of immune surveillance.

## MATERIALS AND METHODS

### Ethics statement.

All animal experiments described here were performed in strict accordance with protocols reviewed and approved by the UConn Health Center Institutional Animal Care and Use Committee (Animal Welfare Assurance no. A347-01) and followed the recommendations of the *Guide for the Care and Use of Laboratory Animals* of the National Institutes of Health (71).

### Bacterial strains and culture conditions.

TOP10 and Stellar *E. coli* strains were grown in Luria-Bertani (LB) broth or on LB plates with appropriate antibiotics (kanamycin [Kan; 50 μg/ml], ampicillin [Amp; 100 μg/ml], and spectinomycin [Spec; 100 μg/ml]) at 37°C. The B31 Δ*flaB* mutant (MC-1; Kan^r^) (45) was generously provided by Nyles Charon (University of West Virginia). All other *B. burgdorferi* strains used in this study are derivatives of B31 5A18 NP1 (72). *B. burgdorferi* strains were routinely cultivated in modified Barbour-Stoenner-Kelly II (BSK-II) medium (73) supplemented with 6% rabbit serum (Pel-Freeze BioLogicals, Rogers, AR) and appropriate antibiotics (kanamycin [Kan; 400 μg/ml], streptomycin [Strep; 50 μg/ml], and/or erythromycin [Erm; 0.06 μg/ml]). Media used for experiments involving BbAG132 also contained specified concentrations of isopropyl-β-d-thiogalactoside (IPTG; see below). Spirochetes were cultivated from mouse tissues as previously described (74). All strains were evaluated for plasmid content as previously described (75).

### Sequence alignments and phylogeny.

Multiple-sequence alignments of the five *Bb*OppA proteins were generated using MUSCLE (78) and displayed with ESPript 3.0 (76). The MUSCLE output file was submitted to PhyML program (79) for phylogenetic analysis and the output phylogenetic tree generated was displayed using the Interactive Tree of Life (iTOL) (80).

### Comparison of the *Bb*OppA4 structure with liganded and unliganded Gram-negative and Gram-positive OBPs.

Crystal structures for the OBPs described here were obtained from the RCSB Protein Data Bank (PDB) (81). The structure for *B. burgdorferi* strain B31 OppA4 (PDB ID: 4GL8) was solved at 2.2-Å resolution by the Seattle Structural Genomics Center for Infectious Disease (SSGCID) using crystals generated by the vapor diffusion sitting drop method (39). Unliganded *E. coli* OppA (*Ec*OppA; PDB ID: 3TCH), solved at a resolution of 1.98 Å (9), and *S. enterica* OppA (*Se*OppA; PDB ID: 1RKM), solved at a resolution of 2.4 Å (82), were used to delineate the structural characteristics of Gram-negative OBPs in the open conformation. The structures of *Ec*OppA liganded to a Lys-Gly-Glu tripeptide (PDB ID: 3TCG), solved at resolution of 2.0 Å (9), and *Se*OppA liganded to a Lys-Leu-Lys tripeptide (PDB ID: 1B9J), solved at a resolution of 1.8 Å (83), were used for comparisons of the closed states. *L. lactis* OppA (*Ll*OppA, PDB ID: 3FTO), solved at a resolution of 2.38 Å (84), was used to delineate the structural characteristics of a Gram-positive OBP in the open conformation. The structure for *Ll*OppA (PDB ID: 3DRG) containing a bradykinin nonapeptide (Arg-Pro-Pro-Gly-Phe-Ser-Pro-Phe-Arg), solved at a resolution of 2.5 Å (40), was used for comparison of the closed states.

### Structural homology modeling of *B. burgdorferi* OBPs.

SWISS-MODEL (85) was used to generate closed homology models for *B. burgdorferi* strain B31 OppA1 (BB0328), OppA2 (BB0329), OppA3 (BB0330), and OppA5 (BBA34) using liganded *Bb*OppA4 (PDB ID: 4GL8) as a template. Open homology models for *B. burgdorferi* OBPs were generated using unliganded *Ec*OppA (PDB ID: 3TCH) as a template. All homology models were visualized in UCSF-Chimera (86). Ligand binding sites were visualized in PyMOL (Delano Scientific). All reported RMSD values reflect Cα deviation.

### **Surface electrostatics representations of** *B. burgdorferi* **OBPs.**

To calculate surface charge, PDB files were converted to PQR files using the PDB2PQR server (87) and entered into the APBS Web server (88) for electrostatic calculations. The resulting electrostatic potential maps were visualized using the electrostatic surface coloring tool in UCSF-Chimera (86). Color saturation was reached at −5 *kT*/*e* to +5 *kT*/*e* (*k* = Boltzmann constant; *T* = temperature; *e* = charge on an electron). The default temperature for APBS is 298.15 K. Charges were assigned on each atom using the PDB2PQR server, which sets the pH value at 7.0. Final electrostatic potential maps were colored at a range of −7 *kT*/*e* to +7 *kT*/*e*.

### Volume calculations for *B. burgdorferi* OBP ligand binding cavities.

The CASTp server (89) was used to calculate binding cavity volumes using the closed structures of *Bb*OppA4, *Ec*OppA, and *Ll*OppA and the closed homology models for the other *B. burgdorferi* OBPs. Cavity volumes were determined using a minimum solvent probe radius of 1.4 Å; the resulting images were visualized as cavities in UCSF-Chimera (86). The cavities of liganded *Ec*OppA, *Ll*OppA, and *Bb*OppA4 are displayed as water filled.

### Evolutionary conservation of *B. burgdorferi* OBPs by ConSurf analysis.

The ConSurf Web server (consurf.tau.ac.il) (90) was used to project the evolutionary conservation of AA residues from *B. burgdorferi* B31 OppA1 to OppA5 onto the structure of *Bb*OppA4. ConSurf employs an empirical Bayesian algorithm to determine position-specific evolutionary conservation scores. These conservation scores are converted into a discrete visual scale ranging from the most variable positions (grades 1 to 4) to the intermediately conserved positions (grades 5 and 6) and the most conserved positions (grades 7 to 9) and visualized in UCSF-Chimera.

### Larval acquisition of *B. burgdorferi* and generation of fed nymphs for RNA extractions.

Five-to-eight-week-old female C3H/HeJ mice (Jackson Laboratories, Bar Harbor, ME) were inoculated intradermally with 1 × 10^4^ temperature-shifted (23°C to 37°C) B31 5A18 NP1 spirochetes. At 3 weeks postinoculation, infected mice were used as a blood meal source for pathogen-free larvae (Oklahoma State University, Stillwater, OK) (200 to 300 per mouse). Following drop-off, replete larvae were allowed to molt to nymphs over supersaturated potassium sulfate in an environmental incubator. Twenty infected flat nymphs were applied to each naive C3H/HeJ mouse via the capsule feeding method (91) and allowed to feed to repletion. Nymphs were collected at drop-off for RNA extraction. Transmission of spirochetes to mice was confirmed 2 weeks postinfestation via ear culture and serology.

### Generation of mammalian-host-adapted spirochetes in DMCs.

Mammalian-host-adapted spirochetes for RNA were generated by cultivation in DMCs implanted into the peritoneal cavities of female Sprague-Dawley rats (175 to 200 g) and evaluated for host adaptation as previously described (92). To assess peptide requirements for growth within the mammalian host, wild-type B31 5A18 NP1 and BbAG132 spirochetes were cultivated at 37°C in BSK-II without and with 1 mM IPTG, respectively, and used for routine DMC implants as noted above. BbAG132 cultures were washed in phosphate-buffered saline (PBS) and resuspended in BSK-II prior to implantation to remove IPTG. To assess the morphology of peptide-starved mammalian-host-adapted spirochetes, DMCs were implanted with unwashed NBD mutant spirochetes at 1 × 10^5^ spirochetes/ml and incubated for 1 week. Three days prior to implantation, rats were given water alone or IPTG-treated water (2% sucrose solution containing 80 mM IPTG) (93) and were then maintained under the same condition for the remainder of the experiment.

### Transcriptional analysis of *opp* genes.

Total RNA was isolated as previously described (91) from samples of 200 replete larvae, 150 flat nymphs, 20 replete nymphs, and ~5 × 10^7^ DMC-cultivated spirochetes. cDNAs, prepared with and without reverse transcriptase, were assayed for *opp* gene transcripts using the primer pairs listed in Table S2 in the supplemental material. Optimized amplification conditions for each gene were determined using SsoAdvanced Universal SYBR mix (Bio-Rad, Hercules, CA). Expression of *opp* genes was determined using a TaqMan-based assay and SsoAdvanced Universal Probe Mix (Bio-Rad) and normalized to *flaB* transcripts (94). All assays were performed in quadruplicate with three biological replicates. Internal standards for each assay were generated by cloning the corresponding amplicon into pCR-TOPO 2.1 (Invitrogen) using designated gene-specific primers (Table S2) according to the manufacturer’s instructions.

10.1128/mBio.02047-17.7 TABLE S2  Oligonucleotide primers used in this study. Download TABLE S2, DOCX file, 0.03 MB. Copyright © 2017 Groshong et al. 2017 Groshong et al. This content is distributed under the terms of the Creative Commons Attribution 4.0 International license.

### Construction of *Bb*AG132, a conditional NBD mutant (see Table S2 for primers). (i) Construction of pEcAG137, a shuttle vector containing an IPTG-inducible NBD.

We first constructed IPTG-inducible *bb0334–35* (*oppDF*) in *E. coli-B. burgdorferi* shuttle vector pJSB275 containing PflgB*-aadA* (Strep^r^) (95) using an InFusion HD EcoDry cloning kit (Clontech, Mountain View, CA). The *bb0334–35* insertion was generated by amplification with CloneAmp hi-fi PCR Premix (Clontech, Mountain View, CA) using B31 5A18 NP1 genomic DNA as a template and primers 5′ bb0334-35ind and 3′ bb0334-35ind. The *bb0334–35* amplicon was then inserted into pJSB275 linearized by digestion with NdeI and HindIII (NEB, Ipswich, MA) by InFusion cloning according to the manufacturer’s instruction. The resulting construct, p*Ec*AG137, was confirmed by sequencing using primers 5′ pJSB275 seq and 3′ pJSB275 seq.

### (ii) Construction of pEcAG138, a suicide vector containing an NBD null cassette.

To replace the endogenous NBD genes (*oppDF*), we constructed a suicide vector containing a *bb0334–35* null cassette. The upstream (984-bp) and downstream (992-bp) fragments for *bb0334–35* and the *ermC* (Erm^r^) gene were amplified from B31 5A18 NP1 and pGK12 (96), respectively, with CloneAmp hi-fi PCR Premix using primers 5′ bb0334-35 null F1 and 3′ bb0334-35 null F1 (upstream fragment); 5′ bb0334-35 null F2 and 3′ bb0334-35 null F2 (downstream fragment); and 5′ bb0334-35 null Erm and 3′ bb0334-35 null Erm (*ermC*). All three fragments were combined with BamHI-digested pUC19 (Invitrogen, Carlsbad, CA) using an InFusion HD EcoDry cloning kit. The resulting construct, p*Ec*AG138, was confirmed by sequencing using primers M13F and M13R.

### (iii) Construction of the NBD mutant in *B. burgdorferi*.

Electrocompetent B31 5A18 NP1 was transformed with 4 μg of p*Ec*AG137, and transformants were recovered in BSK-II under selection with Strep and Kan. Recovered clones were screened first by PCR for the Strep^r^ gene (pless Strep F and pless Strep R) and then evaluated for plasmid content. A positive clone with wild-type plasmid content (*Bb*AG128) was further confirmed to carry pEcAG137 by demonstrating recovery of the plasmid following transformation of total genomic DNA from BbAG128 into *E. coli* TOP10 recovery. Electrocompetent BbAG128 was subsequently transformed with 4 μg of pEcAG138 and recovered overnight in BSK-II containing 1 mM IPTG. The following day, transformants were plated in BSK-II with Kan, Strep, Erm, and 1 mM IPTG. Recovered clones were first screened for Erm^r^ by PCR (pless Erm F and pless Erm R); Erm^r^-positive clones were subsequently screened using *bb0334* and *bb0335* flanking primers 5′ bb0334-35 null F1 and 3′ bb0334-35 null F2. A positive clone with wild-type plasmid content (*Bb*AG132) was further confirmed to carry p*Ec*AG137 by demonstrating recovery of the plasmid following transformation of total genomic DNA into *E. coli* TOP10 recovery.

### Growth curves.

*Bb*AG132 cultures were washed and inoculated into 15 ml of BSK-II (approximately 1 × 10^3^ or 1 × 10^6^ spirochetes/ml) with IPTG at concentrations ranging from 0 to 1 mM and incubated for up to 7 days at 37°C. For the recovery experiment (Fig. 4D), *Bb*AG132 cultures were washed and inoculated into 15 ml of BSK-II (approximately 1 × 10^6^ spirochetes/ml) without IPTG. Each day (starting at day 0), 1 mM IPTG was added to 15-ml aliquots of washed *Bb*AG132 and the reaction mixtures were incubated up to 5 days at 37°C. In all experiments, spirochetes (triplicate samples) were enumerated daily by dark-field microscopy using a Petroff-Hausser counting chamber (Hausser Scientific, Horsham, PA).

### Evaluation of spirochete morphology and motility by light microscopy.

Dark-field microscopy was performed on an Olympus BX41 microscope equipped with a Retiga Exi camera (QImaging, Surrey, British Columbia, Canada); images were acquired using a 40× or a 100× oil objective with QCapture software (v2.1; QImaging). All images were processed with ImageJ v1.50i (97). Videos (see Movies S1, S2, and S3 in the supplemental material) of spirochetes in BSK-II were acquired with a 40× objective for 20 frames for a total duration of 2.755 s (0.13775 s per frame). Video frame rates were slowed to 10 frames/s using AVI FR Changer v1.10 (Inmatrix; http://inmatrix.com/files/avifrate_download.shtml). Videos can be viewed using Windows Media Player or VLC media player for Mac OS X (available from http://www.videolan.org/vlc/download-macosx.html).

### Measurement of spirochete length.

*Bb*AG132 cultures were washed and inoculated into 15 ml of BSK-II (1 × 10^6^ spirochetes/ml) with 0 or 1 mM IPTG and incubated for 5 days at 37°C. Images (magnification of ×400) were acquired for samples daily. A minimum of 100 spirochete lengths for each sample on each day of observation were determined using the Metamorph (v7.8.2.0; Molecular Devices, LLC) integrated morphometry analysis tool, and the data were graphed as whisker plots using Prism software (v5.00; GraphPad Software, Inc., San Diego, CA).

### Transmission electron microscopy.

Samples were prepared for transmission electron microscopy as previously described (98). BbAG132 was cultured in BSK-II–1 mM IPTG to late-logarithmic growth and washed twice with PBS. Spirochetes were then resuspended and incubated in BSK-II with or without IPTG. After 7 days, spirochetes were washed in PBS and incubated overnight in 0.1 M sodium cacodylate (CAC) buffer containing 4% paraformaldehyde and 2.5% glutaraldehyde. Samples were then washed in CAC and centrifuged. The pellet was rinsed with fresh CAC and postfixed with 1% OsO_4_–0.8% potassium ferricyanide–CAC, followed by *en bloc* staining with 1% uranyl acetate–distilled water, dehydration in ascending ethanol solutions, and embedding in PolyBed812 (Polysciences, Warminster, PA). Ultrathin (70-nm-thick) sections were cut on an ultramicrotome (Leica), collected onto formvar-coated 200-mesh copper grids (Electron Microscopy Sciences, Hatfield, PA), stained with 6% uranyl acetate–50% methanol, washed, and then stained with 0.4% lead citrate. The stained sections were viewed on a Hitachi H-7650 transmission electron microscope at 80 kV of accelerating voltage. Images were acquired using an AMT camera and Image Capture Engine v6.01 software (Advanced Microscopy Techniques) and processed using ImageJ v.1.42 software (97).

### Virulence testing of the conditional NBD mutant.

Five-to-eight-week-old female C3H/HeJ mice (Jackson Laboratories, Bar Harbor, ME) were inoculated intradermally with 1 × 10^4^ temperature-shifted spirochetes. Testing of the NBD mutant was done in two independent experiments consisting of two cohorts of four mice each. Beginning 3 days prior to inoculation, cohorts of mice were given water alone or IPTG-treated water (2% sucrose solution containing 80 mM IPTG) (93) and then maintained on the same regimen throughout the duration of the study (93). Two weeks postinoculation, infection was evaluated by serology and tissues (inoculation site skin, ear, tibiotarsal joint, bladder, and heart) were collected for culture in BSK-II containing 1 mM IPTG.

### Statistics.

Levels of expression of individual *opp* genes under the different experimental conditions and with the different spirochete lengths were compared using Prism software using the unpaired *t* test with two-tailed *P* values and a 95% confidence interval.
