# Supplementary figures and images for: Peptide Uptake Is Essential for Borrelia burgdorferi Viability and Involves Structural and Regulatory Complexity of its Oligopeptide Transporter
Source: mBio. 2017 Dec 19;8(6):e02047-17. doi: 10.1128/mBio.02047-17 (PMC5736914; doi:10.1128/mBio.02047-17)

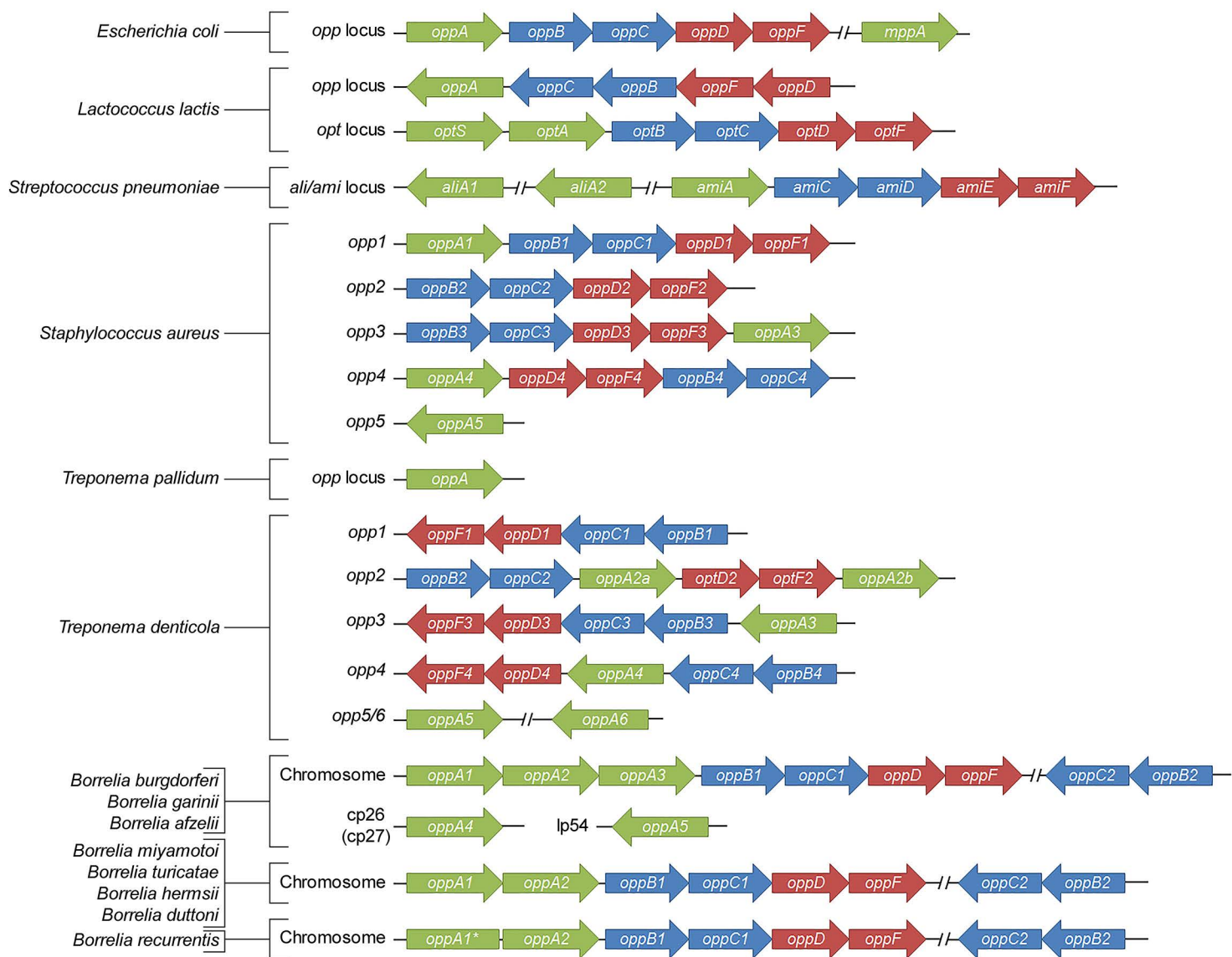

Supplement: FIG S1 [file mbo006173646sf1.pdf]

A

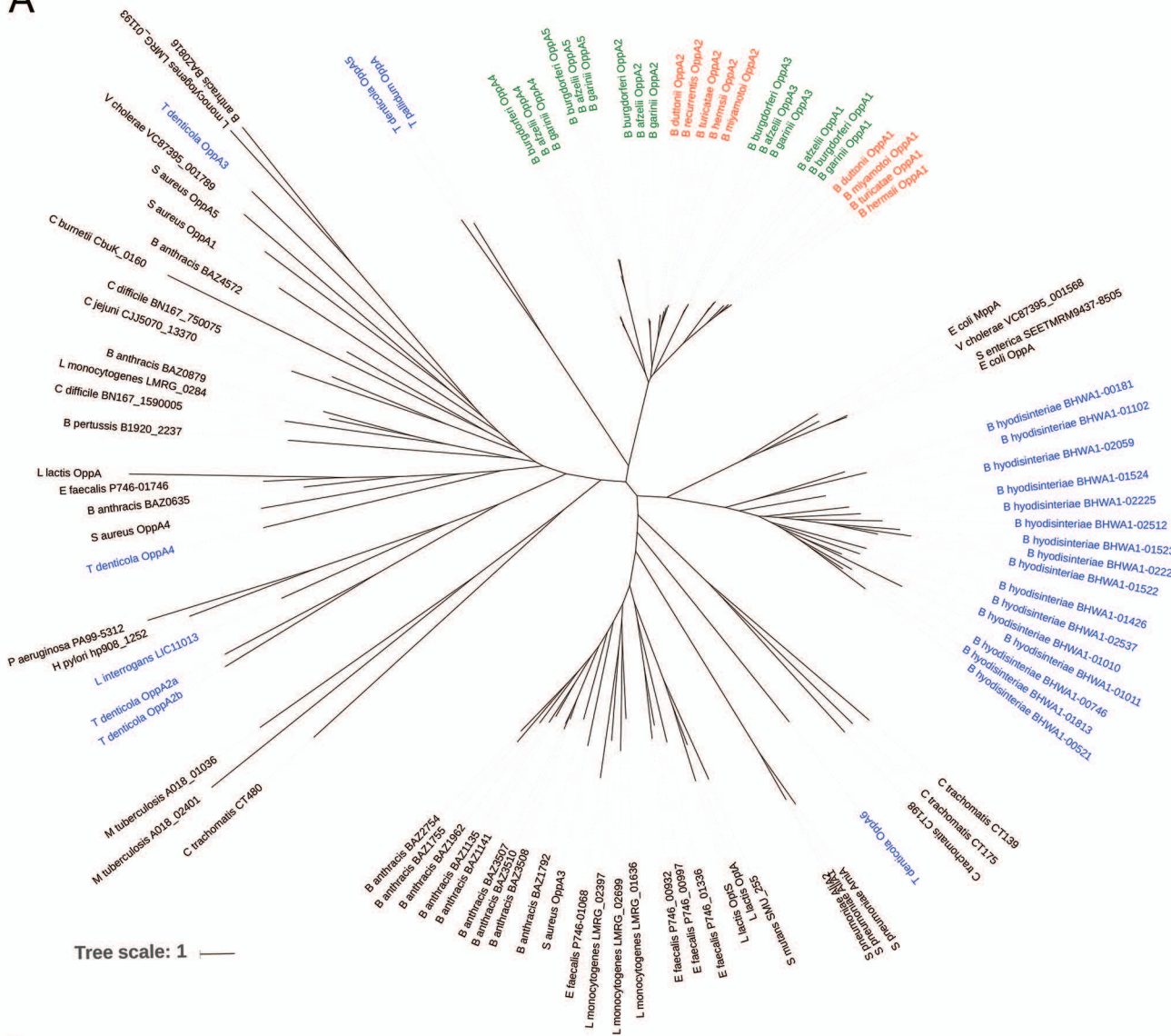

B

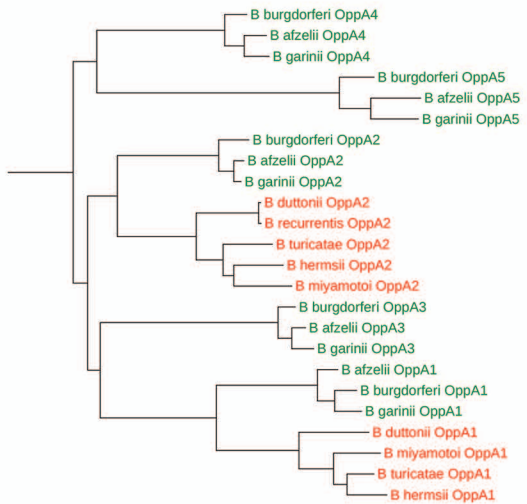

Supplement: FIG S2 [file mbo006173646sf2.pdf]

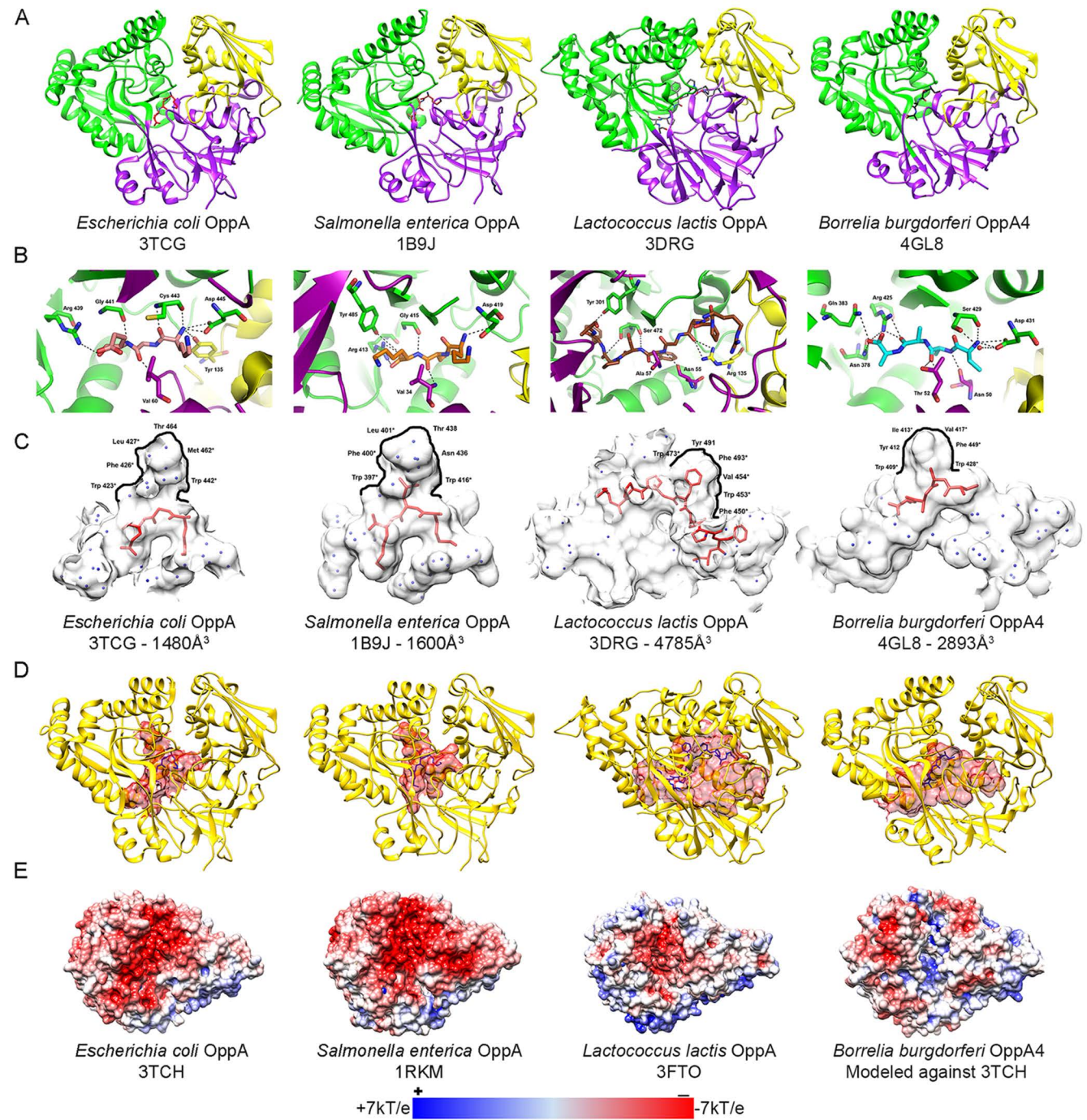

Supplement: FIG S3 [file mbo006173646sf3.pdf]

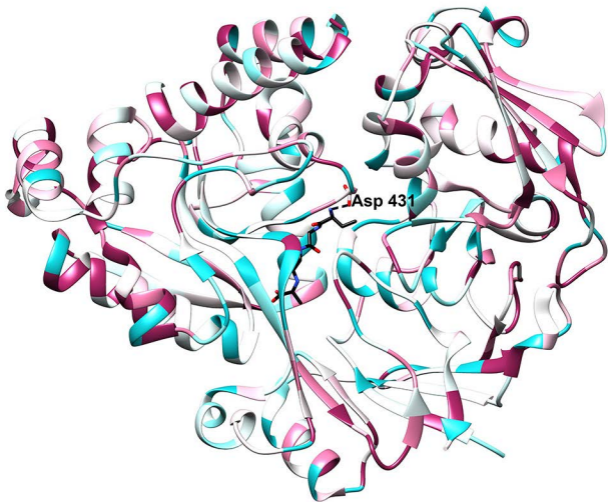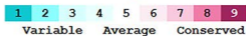

Supplement: FIG S5 [file mbo006173646sf5.pdf]
