# Supplementary material for: Peptide Uptake Is Essential for Borrelia burgdorferi Viability and Involves Structural and Regulatory Complexity of its Oligopeptide Transporter
Source: mBio. 2017 Dec 19;8(6):e02047-17. doi: 10.1128/mBio.02047-17 (PMC5736914; doi:10.1128/mBio.02047-17)
Supplement: TABLE S1 [file mbo006173646st1.docx]

**Supplemental Table 1. *p* value matrix for expression using two-tailed *t* test**

| ***p* value matrix for Fed Larvae transcripts** | | | | | | | | | | | |
| --- | --- | --- | --- | --- | --- | --- | --- | --- | --- | --- | --- |
|  | A1 | A2 | A3 | A4 | A5 | B1 | C1 | B2 | C2 | D | F |
| A1 | - | 0.0588 | 0.0285 | 0.0003 | < 0.0001 | < 0.0001 | < 0.0001 | < 0.0001 | < 0.0001 | < 0.0001 | < 0.0001 |
|  | A2 | - | 0.0146 | 0.0037 | 0.0002 | 0.0003 | 0.0003 | 0.0002 | 0.0002 | 0.0003 | 0.0002 |
|  |  | A3 | - | 0.1681 | 0.0004 | 0.0006 | 0.0007 | 0.0005 | 0.0005 | 0.0008 | 0.0005 |
|  |  |  | A4 | - | < 0.0001 | < 0.0001 | < 0.0001 | < 0.0001 | < 0.0001 | < 0.0001 | < 0.0001 |
|  |  |  |  | A5 | - | < 0.0001 | 0.0001 | 0.0097 | 0.0110 | < 0.0001 | 0.0006 |
|  |  |  |  |  | B1 | - | 0.0292 | < 0.0001 | 0.0044 | 0.0025 | 0.0001 |
|  |  |  |  |  |  | C1 | - | 0.0002 | 0.0020 | 0.0331 | 0.0003 |
|  |  |  |  |  |  |  | B2 | - | 0.0190 | < 0.0001 | 0.0011 |
|  |  |  |  |  |  |  |  | C2 | - | 0.0006 | 0.1548 |
|  |  |  |  |  |  |  |  |  | D | - | 0.0002 |
|  |  |  |  |  |  |  |  |  |  | F | - |
| ***p* value matrix for Flat Nymph transcripts** | | | | | | | | | | | |
|  | A1 | A2 | A3 | A4 | A5 | B1 | C1 | B2 | C2 | D | F |
| A1 | - | 0.0141 | 0.2816 | 0.0093 | 0.0097 | 0.0071 | 0.0072 | 0.0080 | 0.0067 | 0.0215 | 0.0063 |
|  | A2 | - | 0.0107 | 0.0064 | 0.0064 | 0.0061 | 0.0061 | 0.0062 | 0.0060 | 0.0074 | 0.0060 |
|  |  | A3 | - | 0.0031 | 0.0040 | 0.0022 | 0.0022 | 0.0027 | 0.0020 | 0.0148 | 0.0017 |
|  |  |  | A4 | - | 0.7860 | 0.0368 | 0.0218 | 0.1602 | 0.0095 | 0.0303 | 0.0005 |
|  |  |  |  | A5 | - | 0.2531 | 0.2896 | 0.5526 | 0.1744 | 0.0528 | 0.1073 |
|  |  |  |  |  | B1 | - | 0.7766 | 0.3948 | 0.7451 | 0.0121 | 0.4080 |
|  |  |  |  |  |  | C1 | - | 0.4762 | 0.4788 | 0.0118 | 0.1664 |
|  |  |  |  |  |  |  | B2 | - | 0.2272 | 0.0200 | 0.0949 |
|  |  |  |  |  |  |  |  | C2 | - | 0.0089 | 0.5395 |
|  |  |  |  |  |  |  |  |  | D | - | 0.0063 |
|  |  |  |  |  |  |  |  |  |  | F | - |
| ***p* value matrix for Fed Nymph transcripts** | | | | | | | | | | | |
|  | A1 | A2 | A3 | A4 | A5 | B1 | C1 | B2 | C2 | D | F |
| A1 | - | 0.3863 | 0.3389 | 0.0720 | 0.0032 | 0.0034 | 0.0036 | 0.0026 | 0.0038 | 0.0046 | 0.0025 |
|  | A2 | - | 0.1769 | 0.0740 | 0.0133 | 0.0142 | 0.0147 | 0.0120 | 0.0154 | 0.0166 | 0.0118 |
|  |  | A3 | - | 0.6519 | 0.0609 | 0.0676 | 0.0719 | 0.0518 | 0.0769 | 0.0860 | 0.0506 |
|  |  |  | A4 | - | 0.0106 | 0.0119 | 0.0131 | 0.0072 | 0.0143 | 0.0198 | 0.0069 |
|  |  |  |  | A5 | - | 0.5804 | 0.4157 | 0.4274 | 0.2428 | 0.2471 | 0.3595 |
|  |  |  |  |  | B1 | - | 0.7390 | 0.1043 | 0.4352 | 0.3926 | 0.0856 |
|  |  |  |  |  |  | C1 | - | 0.0619 | 0.6616 | 0.5298 | 0.0518 |
|  |  |  |  |  |  |  | B2 | - | 0.0183 | 0.0702 | 0.6101 |
|  |  |  |  |  |  |  |  | C2 | - | 0.7216 | 0.0158 |
|  |  |  |  |  |  |  |  |  | D | - | 0.0619 |
|  |  |  |  |  |  |  |  |  |  | F | - |
| ***p* value matrix for DMC transcripts** | | | | | | | | | | | |
|  | A1 | A2 | A3 | A4 | A5 | B1 | C1 | B2 | C2 | D | F |
| A1 | - | 0.0978 | 0.0783 | 0.3491 | 0.1731 | 0.4758 | 0.3618 | 0.0560 | 0.0499 | 0.7948 | 0.0722 |
|  | A2 | - | 0.0228 | 0.0443 | 0.3407 | 0.0558 | 0.0463 | 0.0198 | 0.0189 | 0.1254 | 0.0219 |
|  |  | A3 | - | 0.1761 | 0.0098 | 0.2457 | 0.2655 | 0.0345 | 0.0010 | 0.0632 | 0.5822 |
|  |  |  | A4 | - | 0.0402 | 0.8755 | 0.9651 | 0.9651 | 0.0842 | 0.2559 | 0.1551 |
|  |  |  |  | A5 | - | 0.0678 | 0.0468 | 0.0079 | 0.0073 | 0.2589 | 0.0096 |
|  |  |  |  |  | B1 | - | 0.8554 | 0.1648 | 0.1441 | 0.3577 | 0.2187 |
|  |  |  |  |  |  | C1 | - | 0.1665 | 0.1420 | 0.2687 | 0.2329 |
|  |  |  |  |  |  |  | B2 | - | 0.3797 | 0.0468 | 0.4186 |
|  |  |  |  |  |  |  |  | C2 | - | 0.0422 | 0.2146 |
|  |  |  |  |  |  |  |  |  | D | - | 0.0587 |
|  |  |  |  |  |  |  |  |  |  | F | - |

*p* values < 0.05 are in green and ≥ 0.05 are in red.
