# Supplementary material for: Peptide Uptake Is Essential for Borrelia burgdorferi Viability and Involves Structural and Regulatory Complexity of its Oligopeptide Transporter
Source: mBio. 2017 Dec 19;8(6):e02047-17. doi: 10.1128/mBio.02047-17 (PMC5736914; doi:10.1128/mBio.02047-17)
Supplement: TABLE S2 [file mbo006173646st2.docx]

**Supplemental Table 2. Oligonucleotide primers used in this study**

| **Designation** | **Sequence (5’-3’)** | **Purpose** | **Reference** |
| --- | --- | --- | --- |
| pless Strep F | ATGAGGGAAGCGGTGATCGCCGA | Confirmation | This study |
| pless Strep R | TTATTTGCCGACTACCTTGGTG | Confirmation | This study |
| pless Erm F | CTTTGAAATCGGCTCAGGAAAAGGCC | Confirmation | This study |
| pless Erm R | TCTGCCATTAAAAGTAATGCCAATGAGCG | Confirmation | This study |
| 5’ pJSB275 seq | GATTCAATTGTGAGCGGAATAACA | pJSB275 seq | This study |
| 3’ pJSB275 seq | ATGCGCTTAACGGTAAAATCCAAGG | pJSB275 seq | This study |
| 5’ bb0334-35 ind | GGAGAAATTACATATGGAAAAAGAAAATATATTGGAAATAAAAAATTTAA | i*bb0334-35* cloning | This study |
| 3’ bb0334-35 ind | CTCTATCTTCAAGCTTTTATCTGTTCATCTCATCAAAAAGGTATTTAGAT | i*bb0334-35* cloning | This study |
| 5’ bb0334-35 null F1 | CGGTACCCGGGGATCCAAGATTTAAAGAAAATAAACTCGCATTTGGCAGT | Δ*bb0334-35* cloning | This study |
| 3’ bb0334-35 null F1 | TAGAGCACACGGTTTTCAATTTCTCCTTAGATGCTATCTTTTGGATCAAA | Δ*bb0334-35* cloning | This study |
| 5’ bb0334-35 null erm | CTAAGGAGAAATTGAAAACCGTGTGCTCTACGACCAAAACTATAAAACCT | Δ*bb0334-35* cloning | This study |
| 3’ bb0334-35 null erm | TGATCATAAGTAAGCGATTCACAAAAAATAGGCACACGAAAAACAAGTTA | Δ*bb0334-35* cloning | This study |
| 5’ bb0334-35 null F2 | TATTTTTTGTGAATCGCTTACTTATGATCAAAATAGGAAACTTCGAAATC | Δ*bb0334-35* cloning | This study |
| 3’ bb0334-35 null F2 | CGACTCTAGAGGATCCGATGGTACGGCAGCTCTTCCGTAAGTTCCATCTT | Δ*bb0334-35* cloning | This study |
| 5’ bb0328 | ATGAAATATATAAAAATAGCCTTAATGCTAATAATTTTTTCTTTAATAGC | qRT standard | This study |
| 3’ bb0328 | TTATTTTTTAGTTTTAATATCTTCATATAAATAGCTTTCTGCGATATTTG | qRT standard | This study |
| 5’ bb0329 | ATGAAATTACAAAGGTCATTATTTTTAATAATATTTTTTCTAACTTTTCT | qRT standard | This study |
| 3’ bb0329 | TTATTTATTTTTTAATTTTAGCTGAGATAAATCAAATCTTTCTAAAATAT | qRT standard | This study |
| 5’ bb0330 | ATGAGCTTTAATAAAACTAAAAAAATCGGTAAAAAAATTAAAATA | qRT standard | This study |
| 3’ bb0330 | TTAATTATGTTTTGCATTTTTAATTGGTTTTAATTCAGAAAGATA | qRT standard | This study |
| 5’ bb0332 | ATGTTAAAGTTTACTTTAAAGAAAATATTAGGAATAATACCAACTTTACT | qRT standard | This study |
| 3’ bb0332 | TTATACTCTTGGATCTAATATTTTATATATAATATCTGATATTAATATAG | qRT standard | This study |
| 5’ bb0333 | ATGAATAGCCTTGAAAAACAAAATGAAGAAAACAA | qRT standard | This study |
| 3’ bb0333 | TTAGATGCTATCTTTTGGATCAAAAGCATCCCTTA | qRT standard | This study |
| 5’ bb0334 | ATGGAAAAAGAAAATATATTGGAAATAAAAAATTTAACAA | qRT standard | This study |
| 3’ bb0334 | TTAAAACTCCTCGGTGCTGGTTTTTGTGAT | qRT standard | This study |
| 5’ bb0335 | ATGAGTAGTAAAAAAGAAATAATTCTTAAAGTAGAAAACTTAATGCAAAC | qRT standard | This study |
| 3’ bb0335 | TTATCTGTTCATCTCATCAAAAAGGTATTTAGATACAAAG | qRT standard | This study |
| 5’ bb0746 | ATGAAAACAGATACAATAATAAAAAAAATTTATATCGTACTCTTTAATAT | qRT standard | This study |
| 3’ bb0746 | TTAAGATGATATTAAATGTTTCATTTTTTTTCTTAGCTTAAAATT | qRT standard | This study |
| 5’ bb0747 | TTGAAAATATTTATTTTTAAAAATACAATATATTTATTAATTAATTTAAT | qRT standard | This study |
| 3’ bb0747 | TTAGTTTAGAGTGTCCTTATATGGGTTAATTTTGTAAATTAGTATATCTG | qRT standard | This study |
| 5’ bbb16 | ATGAAAATATTGATAAAAAAGTTAAAAGTTGTATTATTTCTCAATTTAAT | qRT standard | This study |
| 3’ bbb16 | TCATTTAATTGGTTTTATTTCAGATAAATTAAATCTTTCCGAAATATTTG | qRT standard | This study |
| 5’ bba34 | ATGATAATAAAAAAAAGAGGACTTTTAATACTGGGCATTG | qRT standard | This study |
| 3’ bba34 | TTATTCTTCTATAGGTTTTATTTCTGATAGGGCAAATCTT | qRT standard | This study |
| bb0328 F | GAGCTTGGCATAAAGGCTATTG | qRT-PCR | ^1^ |
| bb0328 R | CTGGTATGTATGCTGAGTGTGT | qRT-PCR | ^1^ |
| bb0329 F | AACGAAGAATGGACAACATACTTAAACA | qRT-PCR | ^1^ |
| bb0329 R | AATGTCAAAGGATCAGCATAATCG | qRT-PCR | ^1^ |
| bb0330 F | CGCATTCATGCCAGTACCTATT | qRT-PCR | ^1^ |
| bb0330 R | AGGACCGCTAGTAACCATGT | qRT-PCR | ^1^ |
| bb0332-575-F | CAAGAATAATCAGAGGATCAATG | qRT-PCR | This study |
| bb0332-759-R | TTCAATAACCACGCTTCC | qRT-PCR | This study |
| bb0333-616-F | ATGGAAAGAAGTATAATCGGCTTA | qRT-PCR | This study |
| bb0333-794-R | ATGCTATTAGGGATCAAGTGTTT | qRT-PCR | This study |
| bb0334-429-F | TGTTGTAAACGCAGAAGAAAGA | qRT-PCR | This study |
| bb0334-561-R | GGCTGTTGTTGGTTCATCT | qRT-PCR | This study |
| bb0335-145-F | TTAGGACTCGTAGGAGAATCTG | qRT-PCR | This study |
| bb0335-335-R | GGGTCAAGCGAAGTATGG | qRT-PCR | This study |
| bb0746-159-F | ACCTATGCCACCCACAAA | qRT-PCR | This study |
| bb0746-276-R | TGTTGCGTAGCTTAGTGAAAG | qRT-PCR | This study |
| bb0747-185-F | CCCTGATACATGACTTTAACCCTA | qRT-PCR | This study |
| bb0747-345-R | TTTAGTAGTTGGCTTTCCTTTGC | qRT-PCR | This study |
| bbb16-F | ACGGTTGGATCAGGGATTGTAAGCCA | qRT-PCR | ^1^ |
| bbb16-R | TCCTTCGGCAGTAATGGAAACTCC | qRT-PCR | ^1^ |
| bba34-F | CAAGCGATGTTGGTTCGTTTC | qRT-PCR | ^1^ |
| bba34-R | TACTGGGCATTGCTACTGTAATC | qRT-PCR | ^1^ |
|  |  |  |  |

**Supplemental References**

1 Iyer, R. *et al.* Stage-specific global alterations in the transcriptomes of Lyme disease spirochetes during tick feeding and following mammalian host adaptation. *Mol Microbiol* **95**, 509-538, doi:10.1111/mmi.12882 (2015).
