# Supplementary material for: Peptide Uptake Is Essential for Borrelia burgdorferi Viability and Involves Structural and Regulatory Complexity of its Oligopeptide Transporter
Source: mBio. 2017 Dec 19;8(6):e02047-17. doi: 10.1128/mBio.02047-17 (PMC5736914; doi:10.1128/mBio.02047-17)
Supplement: FIG S4 [file mbo006173646sf4.pdf]

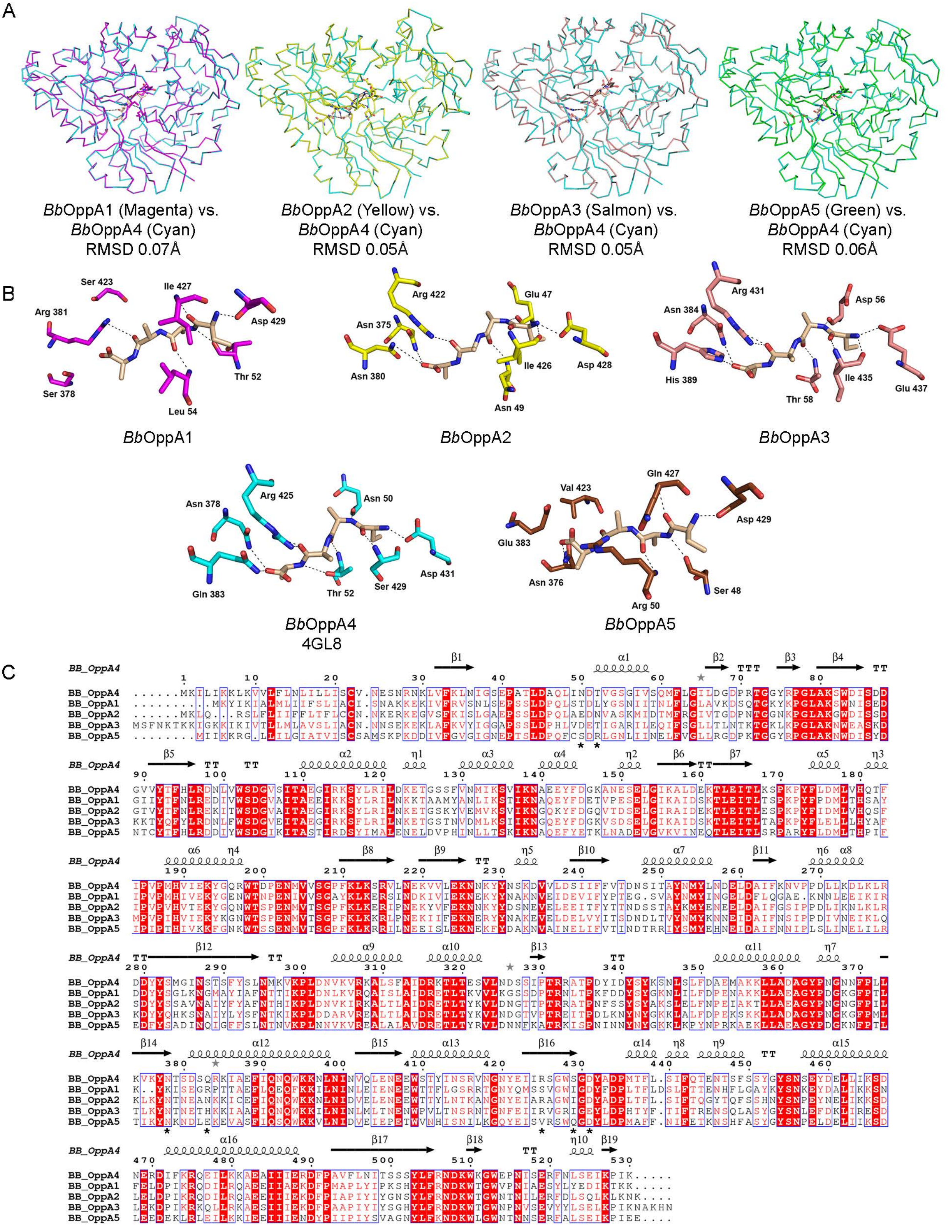
A
BbOppA1 (Magenta) vs.
BbOppA4 (Cyan)
RMSD 0.07Å
BbOppA2 (Yellow) vs.
BbOppA4 (Cyan)
RMSD 0.05Å
BbOppA3 (Salmon) vs.
BbOppA4 (Cyan)
RMSD 0.05Å
BbOppA5 (Green) vs.
BbOppA4 (Cyan)
RMSD 0.06Å
B
Ser 423
Ile 427
Arg 381
Asp 429
Thr 52
Ser 378
Leu 54
BbOppA1
Arg 422
Glu 47
Asn 375
Asn 380
Ile 426
Asp 428
Asn 49
BbOppA2
Arg 431
Asn 384
Asp 56
His 389
Thr 58
Ile 435
Glu 437
BbOppA3
Asn 378
Arg 425
Asn 50
Gln 383
Thr 52
Ser 429
Asp 431
BbOppA4
4GL8
Val 423
Gln 427
Glu 383
Asp 429
Asn 376
Arg 50
Ser 48
BbOppA5
C
BB_OppA4 ......MKILIKKLKVVLFLNLILLISCV.NESNRNKLVFKLNIGSEPATLDAQLINDTVGSGIVSQMFLGILDGDPRTGGYRPGLAKSWDISDD
BB_OppA1 ........MKYIKIALMLIIFSLIACI.SNAKKEKIVFRVSNLSEPSSLDPQLSTDLYGSNIITNLFLGLAVKDSQTGKYKPGLAKSWNISED
BB_OppA2 ......MKLQ...RSLFLIIFFLTFLCCN.NKERKEGVSFKISLGAEPSSLDPQLAEDNVASKMIDTMFRGIVTGDPNTGGNKPGLAKGWDISSD
BB_OppA3 MSFNKTKKIGKKIKIVLTLLMLAVSLIACN.NNSEKEKLAFKVYIGGAPSSLDPHLVDETIGARILEQIFSGLLTLNTKTGKLKPGLAKNWEASKD
BB_OppA5 ....MIIKKRG.LLILGIATVISCSAMSKPKDDIVFGVGIGNEPTSLDPQFCSDRLGNLIINELFVGLLRGDPKTGGYRPGLAKNWDISYD
BB_OppA4 GVVYTFHLRDNLVWSDGVSITAEGIRKSYLRILDKETGSSFVNMIKSVIKNAEEYFDGKANESELGIKALDEKTLEITLKSPKPYFLDMLVHQTF
BB_OppA1 GIIYTFNLREDIVWSDGVAITAEEIKKSYLRILNKKTAAMYANLIKSTIKNAQEYFDETVPESELGIKAIDSKTLEITLTSPKPYFPDMLTHSAY
BB_OppA2 GTVYTFNLREKITWSDGVAITAEGIRKSYLRILNKETGSKYVEMVKSVIKNGQKYFDGQVTDSELGIRAIDEKTLEITLESPKPYFIDMLVHQSF
BB_OppA3 KKTYQFYLRDNLFWSDGVEITAEGIRKSFLRILNKETGSTNVDMLKSIIKNGQEYFDGKVSDSELGIKAIDSKTLEITLTAPKPYFLELLLHYAF
BB_OppA5 NTCYTFHLRDDIYWSDGIKITASTIRDSYIMALENELDVPHINLLTSKIKNAQEFYETKLNADEVGVKVINEQTLEITLSRPARYFLDMLTHPIF
BB_OppA4 IPVPMHVIEKYGQRWTDPENMVVSGPFKLKSRVLNEKVVLEKNNKYYNSKDVVLDSIIFFVTDNSITAYNMYLNDELDAIFKNVPPDLLKDLKLR
BB_OppA1 IPVPMHIVEKYGENWTNPENIVVSGAYKLKERSINDKIVIEKNEKYYNAKNVEIDEVIFYPTEG.SVAYNMYINGELDFLQGAE.KNNLEEIKIR
BB_OppA2 IPVPVHVTEKYGQNWTSPENMVTSGPFKLKERIPNEKYVFEKNNKYYDSNEVELEEITFYTTNDSSTAYKMYENEELDAIFGSIPPDLIKNLKLR
BB_OppA3 MPVPIHVIEKYKGNWTSPENMVTSGPFKLKKRLPNEKIIFEKNERYYNAKEVELDELVYITSDNDLTVYNMYKNNEIDAIFNSIPPDIVNEIKLQ
BB_OppA5 IPIPTHIVKKFGNKWTSSENMVTSGPFKLKRRILNEEISLEKNEKFYDAKNVAINELIFVTINDTRRIYSMYEHNEIDAIFNNIPLSLINELILR
BB_OppA4 DDYYSMGINSTSFYSLNMKVKPLDNVKVRKALSFAIDRKTLTESVLNDSSIPTRRATPDYIDYSYKSNLSLFDAEMAKKLLADAGYPNGNNFPLL
BB_OppA1 DDYYSGLKNGMAYIAFNTTIKPLDNLKVRQAISLAIDRETLTKVVLKGSSDPTRNLTPKFDDYSYGKNLILFDPENAKKLLAEAGYPDGKGFPTL
BB_OppA2 SDYYSSAVNAIYFYAFNTHIKPLDNVKIRKALTLAIDRETLTYKVLDNGTTPTRRATPNFSSYSYAKSLELFNPEIAKTLLAEAGYPNGNGFPIL
BB_OppA3 KDYYQHKSNAIYLYSFNTKIKPLDDARVREALTLAIDRETLTYKVLNDGTVPTREITPDLKNYNYGKKLALFDPEKSKKLLADAGYPNGKGFPML
BB_OppA5 EDFYSADINQIGFFSLNTNVKPLNNVKVREALALAVDRETLTYRVLDNNFKATRKISPNINNYNYGKKLKPYNPRKAEKLLAEAGYPDGKNFPTL
BB_OppA4 KVKYNTSDSQRKIAEFIQNQWKKNLNINVQLENEEWSTYINSRVNGNYEIIRSGWSGDYADPMTFL.SIFQTENTSFSSYGYSNSEYDELLIKSD
BB_OppA1 K..YKISEGRPTTAEFLQEQFKKILNINLEIENEEWTTFLGSRRTGNYQMSSVGWIGDYFDPLTFLDSLFTTENHFLGAYKYSNKEYDALIKKSN
BB_OppA2 KLKYNTNEANKKICEFIQNQWKKNLNIDVELENEEWTTYLNTKANGNYEIARAGWIGDYADPLTFL.SIFTQGYTQFSSHNYSNPEYNELIKKSD
BB_OppA3 TLKYNTNETHKKIAAFIQNQWKKILNINLMLTNENWPVLTNSRNTGNFEIIRVGRIGEYLDPHTYF.TIFTRENSQLASYGYSNLEFDKLIRESD
BB_OppA5 TIKYNKNDLEKEVASFIQSQWKKVLNIDVEIEPETWVNHISNILKGQYEISVRSWQGDYLDPMAFF.NIFETKNSHFASYGYSNPELDELIIKSD
BB_OppA4 NERDIFKRQEILKKAEAIIIERDFPAVFLNITSSSYLFRNDKWKGWEPNISERFNLSEIKPIK.....
BB_OppA1 FELDPIKRQDILRQAEEIIAEKDFPMAPLYIPKSHYLFRNDKWTGWVPNIAESYLYEDIKTKK.....
BB_OppA2 LELDPIKRQDILRQAEEIIIEKDFPIAPIYIYGNSYLFRNDKWTGWNTNILERFDLSQLKLKNK....
BB_OppA3 LEKDPIKRKQLLRKAESIIIEKDFPAAPIYIYSGHYLFRNDKWTGWNPNVSEVYYLSELKPIKNAKHN
BB_OppA5 LEEDEKLRLEILKKIEEIIIENDYPIIPIYSVAGNYLFKNDKWLGWNTNNSERFALSEIKPIEE....
